# Supplementary material for: EPDR1 promotes PD-L1 expression and tumor immune evasion by inhibiting TRIM21-dependent ubiquitylation of IkappaB kinase-β
Source: EMBO J. 2024 Aug 16;43(19):4248–73. doi: 10.1038/s44318-024-00201-6 (PMC11445549; doi:10.1038/s44318-024-00201-6)
Supplement: Supplementary file 2 — Appendix [file 44318_2024_201_MOESM2_ESM.pdf]

EPDR1 promotes PD-L1 expression and tumor immune evasion by inhibiting TRIM21-dependent ubiquitylation of IkappaB kinase-beta

Xiaoyu Qian[1,3], Jin Cai[1,3], Yi Zhang[1], Shengqi Shen[2], Mingjie Wang[1], Shengzhi Liu[1], Xiang Meng[1], Junjiao Zhang[1], Zijian Ye[1], Shiqiao Qiu[1], Xiuying Zhong[2*], Ping Gao[1,2*]

[1] School of Medicine, South China University of Technology, Guangzhou, China

[2] Medical Research Institute, Guangdong Provincial People's Hospital, Guangdong Academy of Medical Sciences, Southern Medical University, Guangzhou, China

[3] These authors contributed equally to this work

[*] Correspondence: pgao2@ustc.edu.cn, zxywawj@ustc.edu.cn

This PDF file includes:

Appendix Figure S1. Neutralization of PD-L1 abolishes the advantage in liver orthotopic xenograft development derived by EPDR1.

Appendix Figure S2. Suppression of EPDR1 attenuates PD-L1-mediated immune evasion of HCC cells.

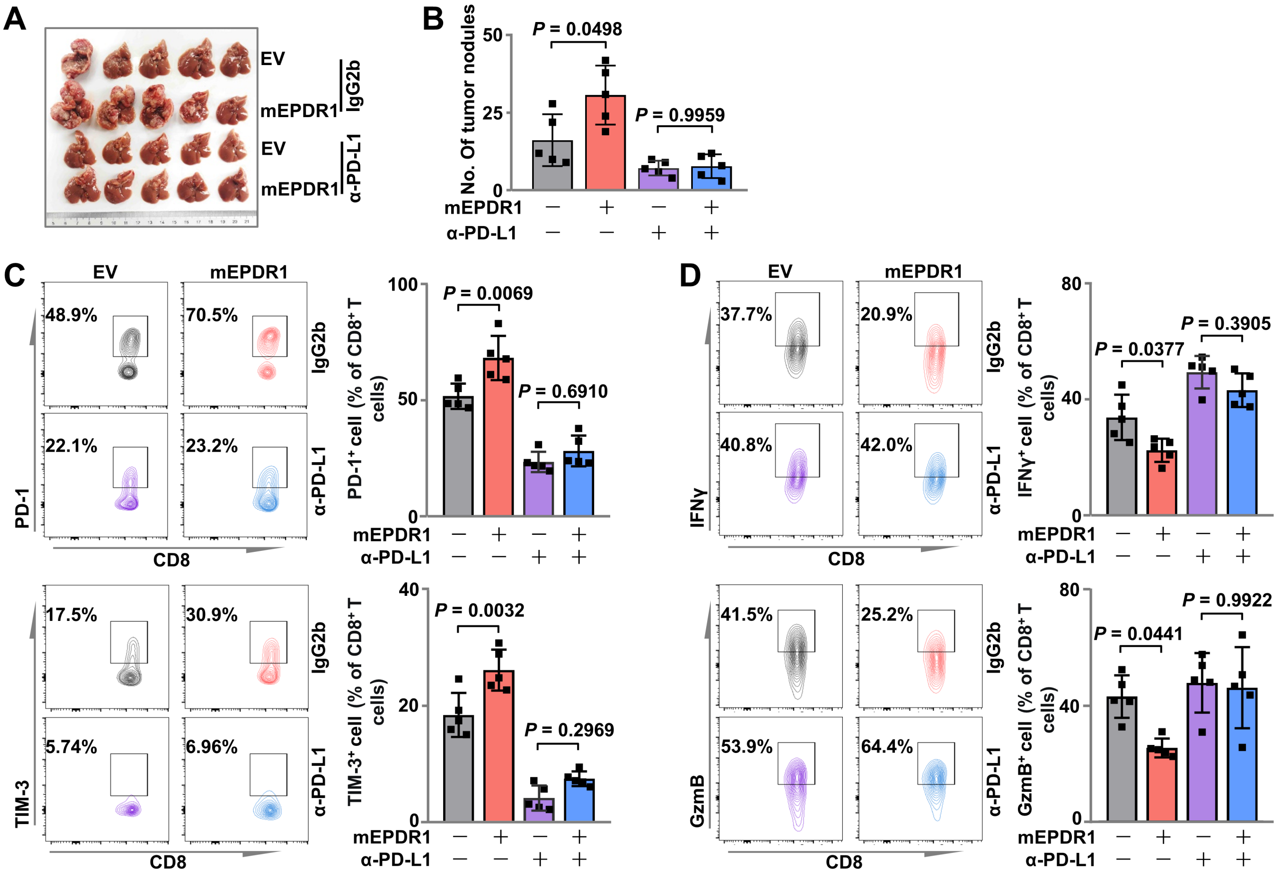

Appendix Figure S1. Neutralization of PD-L1 abolishes the advantage in liver orthotopic xenografts development derived by EPDR1.

A, B Hepa1-6 cells stably expressing Flag-EV and Flag-mEPDR1 were injected via the hepatic portal vein into C57BL/6J mice (n = 5 male mice each group), and α-PD-L1 (4 mg/kg) neutralizing antibody was injected intraperitoneally 4 times (twice a week starting at 10 days after inoculation) to block PD-L1 and IgG2b as a control. Photographs show liver xenografts (A) and tumor numbers (B) determined at the end of the experiment (day 28). Data are presented as the mean ± SD.

C, D Flow cytometry analysis of the ratio of immune co-suppressive molecules (PD-1, TIM-3) and immune effector molecules (IFNγ, GzmB) positive cells in tumor $CD8^+$ T cells from the indicated group in (A). Data are presented as the mean ± SD (n = 5 male mice each group).

Data information: Statistical significance was determined by one-way ANOVA (B-D).

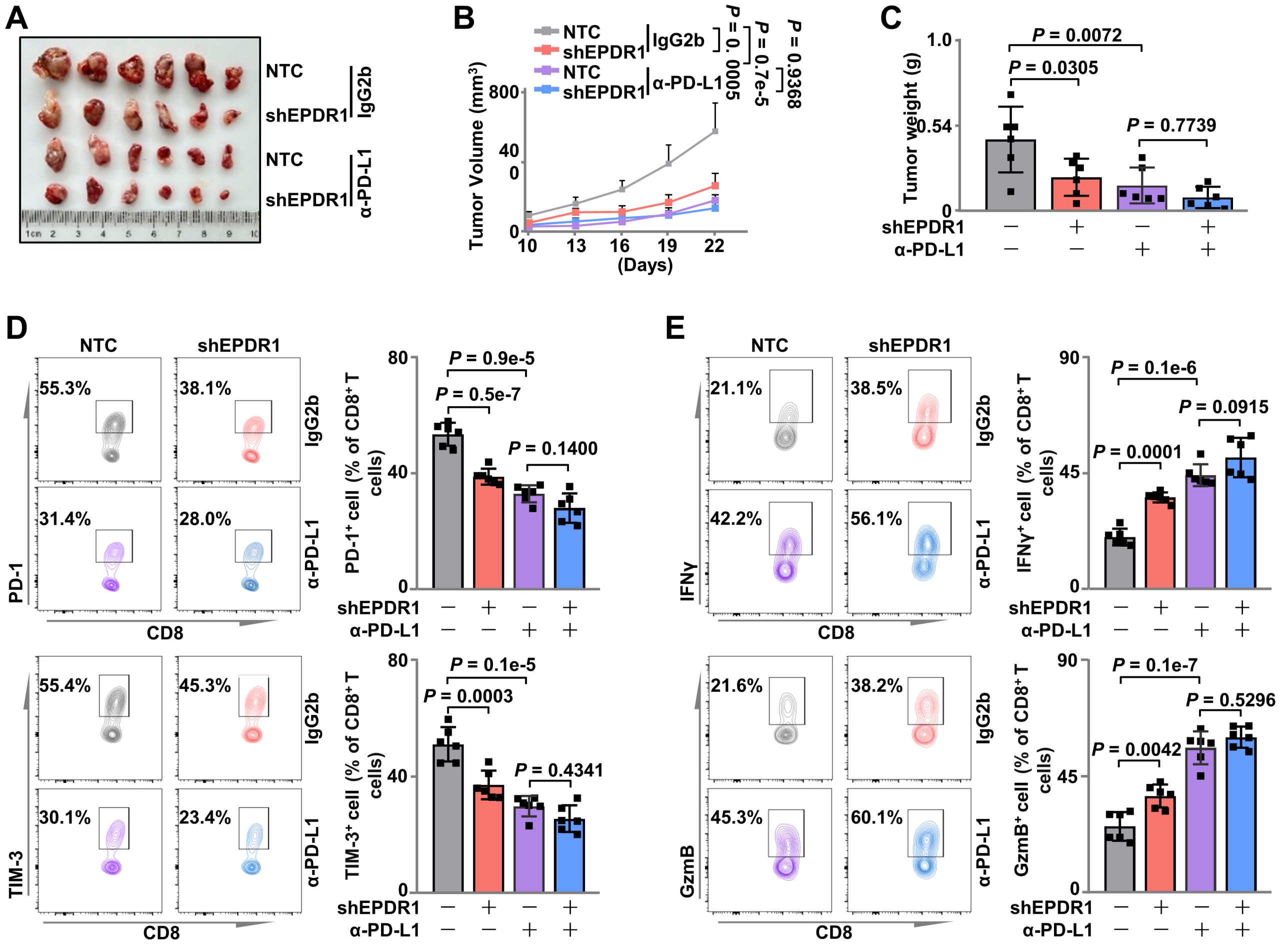

Appendix Figure S2. Suppression of EPDR1 attenuates PD-L1-mediated immune evasion of HCC cells.

A-C Hepa1-6 cells stably expressing NTC or shEPDR1 were injected subcutaneously into C57BL/6J mice (n = 6 male mice per group). Tumor size was measured starting at 10 days after inoculation. Figures depict xenografts (A), growth curves (B) and tumor weight (C) determined at the end of the experiment (day 25). Data are presented as the mean ± SEM.

D Flow cytometric analysis of ratio of immunosuppressive molecules (PD-1, TIM-3) positive cells in tumor $CD8^+$ T cells from the indicated group in (A). Data are presented as the mean ± SD.

E Flow cytometric analysis of ratio of immune effector molecules (IFNγ, GzmB) positive cells in tumor $CD8^+$ T cells from the indicated group in (A). Data are presented as the mean ± SD.

Data information: Statistical significance was determined by two-way ANOVA (B), and one-way ANOVA (C-E).
