# Supplementary material for: EPDR1 promotes PD-L1 expression and tumor immune evasion by inhibiting TRIM21-dependent ubiquitylation of IkappaB kinase-β
Source: EMBO J. 2024 Aug 16;43(19):4248–73. doi: 10.1038/s44318-024-00201-6 (PMC11445549; doi:10.1038/s44318-024-00201-6)
Supplement: Supplementary file 3 — Source data Fig. 1 [file 44318_2024_201_MOESM3_ESM.zip › EMBOJ-2023-116324_SourceDataForFigure1B-G_source_data.pdf]

B

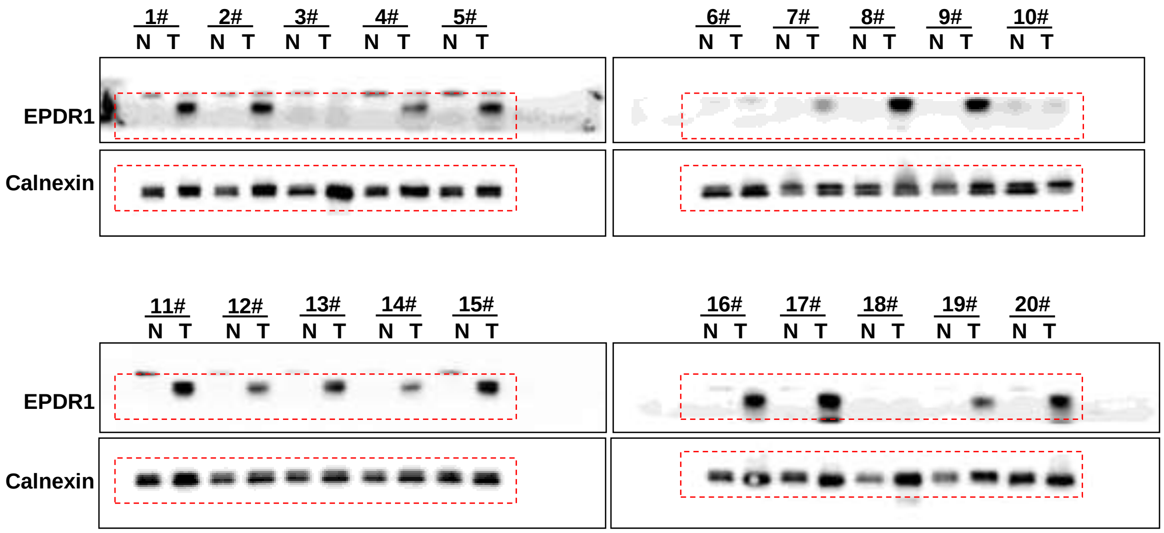

1# 2# 3# 4# 5#
N T N T N T N T N T
6# 7# 8# 9# 10#
N T N T N T N T N T
EPDR1
Calnexin
11# 12# 13# 14# 15#
N T N T N T N T N T
16# 17# 18# 19# 20#
N T N T N T N T N T
EPDR1
Calnexin

C

expression	group
0.810884	noncancerous
1.055237	noncancerous
12.1895	noncancerous
1.888928	noncancerous
0.241077	noncancerous
2.689928	noncancerous
1.326447	noncancerous
1.482023	noncancerous
1.047948	noncancerous
8.861602	noncancerous
0.851199	noncancerous
7.608265	noncancerous
0.527619	noncancerous
0.569421	noncancerous
0.290693	noncancerous
0.153634	noncancerous
0.39986	noncancerous
0.440608	noncancerous
0.482155	noncancerous
0.527619	noncancerous
1.955541	noncancerous
0.42856	noncancerous
0.875129	noncancerous
0.805283	noncancerous
0.343306	noncancerous
0.845319	noncancerous
0.546225	noncancerous
0.43154	noncancerous
0.362881	noncancerous
0.705916	noncancerous
26.31051	tumor
3.75176	tumor
2.125154	tumor
3.288814	tumor
5.26914	tumor
1.07741	tumor
9.302172	tumor
0.951033	tumor
5.969324	tumor
13.15526	tumor
2.544827	tumor
3.549381	tumor
14.80043	tumor
4.523902	tumor
5.26914	tumor
14.49585	tumor
5.846477	tumor
0.79972	tumor
1.774691	tumor
2.984662	tumor
1.402079	tumor
2.139936	tumor
1.787035	tumor
34.71693	tumor
1.326447	tumor
3.884066	tumor
0.751355	tumor
0.899732	tumor
5.019583	tumor
7.822163	tumor

D

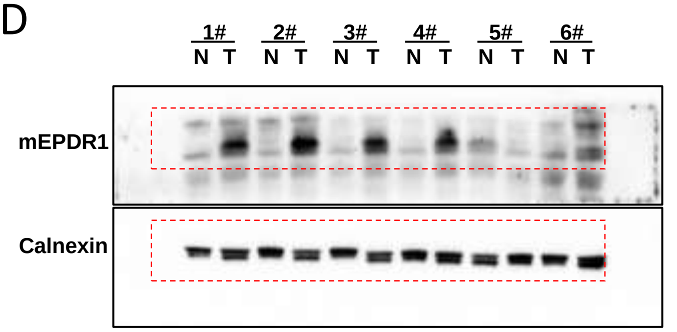

E

expression	group
2.017404	noncancerous
1.216301	noncancerous
0.587435	noncancerous
1.174869	noncancerous
0.625248	noncancerous
0.106764	noncancerous
0.467326	noncancerous
10.87166	noncancerous
1.955449	noncancerous
0.318088	noncancerous
0.670124	noncancerous
0.161824	noncancerous
0.535191	noncancerous
2.728532	noncancerous
0.253161	noncancerous
0.200007	noncancerous
0.1014	noncancerous
0.783565	noncancerous
132.7437	Tumor
147.2884	Tumor
18.53911	Tumor
84.59497	Tumor
125.5832	Tumor
65.91341	Tumor
34.35623	Tumor
164.5634	Tumor
3.440218	Tumor
0.60395	Tumor
1.497442	Tumor
4.732164	Tumor
108.9955	Tumor
22.59803	Tumor
11.94326	Tumor
1.131412	Tumor
23.88652	Tumor
138.9214	Tumor

F

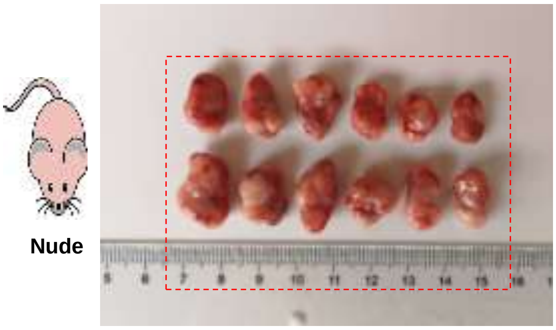

Nude

EV

mEPDR1

	Days	EV						EPDR1						
Tumor volume	10	64.3	97.2	77.1	34.5	50.2	116.5	116.3	61.0	40.5	52.5	123.3	237.3	nude
	13	219.5	241.9	220.9	76.6	84.5	197.7	131.7	403.7	149.7	157.3	319.8	602.7	
	16	534.8	549.6	514.7	140.7	235.3	385.6	884.9	1107.3	343.6	723.1	524.7	261.4	
	19	712.0	999.6	815.8	491.3	582.0	356.7	1341.9	1076.2	1033.0	1078.7	788.2	568.4	
	21	1303.8	1197.3	1184.0	690.5	1021.9	506.6	1702.4	1214.6	1494.6	1554.7	934.5	1007.5	
		EV						EPDR1						
Tumor weight		0.54	0.84	0.83	0.84	0.54	0.54	0.6	0.66	1.21	1.05	0.95	1.3	

G

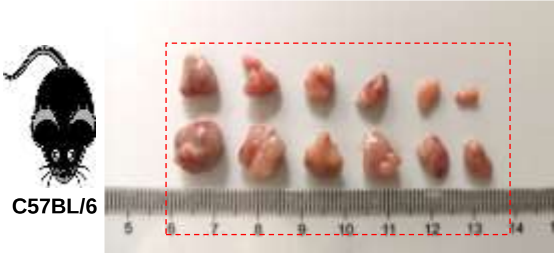

C57BL/6

EV

mEPDR1

	Days	EV						EPDR1						
Tumor volume	10	50.0	31.7	29.0	18.3	15.1	12.7	52.9	58.7	149.4	65.7	18.4	10.8	c57bl/6
	13	87.6	64.3	51.6	35.1	23.0	26.6	229.9	192.3	432.4	72.5	88.0	49.7	
	16	157.9	129.0	76.9	47.8	40.3	30.1	387.4	615.8	316.6	345.9	178.1	141.8	
	19	288.6	258.8	158.7	197.7	45.2	44.5	558.1	765.1	459.7	418.2	281.9	170.9	
	21	317.4	362.8	280.5	219.0	190.2	81.7	901.3	874.0	586.8	519.9	372.7	264.2	
		EV						EPDR1						
Tumor weight		0.15	0.06	0.15	0.04	0.1	0.21	0.47	0.16	0.67	0.33	0.22	0.31	
