# Supplementary material for: EPDR1 promotes PD-L1 expression and tumor immune evasion by inhibiting TRIM21-dependent ubiquitylation of IkappaB kinase-β
Source: EMBO J. 2024 Aug 16;43(19):4248–73. doi: 10.1038/s44318-024-00201-6 (PMC11445549; doi:10.1038/s44318-024-00201-6)
Supplement: Supplementary file 4 — Source data Fig. 2 [file 44318_2024_201_MOESM4_ESM.zip › EMBOJ-2023-116324_SourceDataForFigure2A-H.pdf]

A

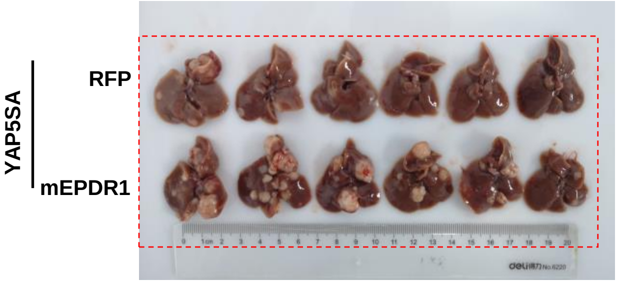

B

No. Of tumor nodules	
RFP	mEPDR1
5	6
3	9
7	12
3	8
1	3
1	6

C

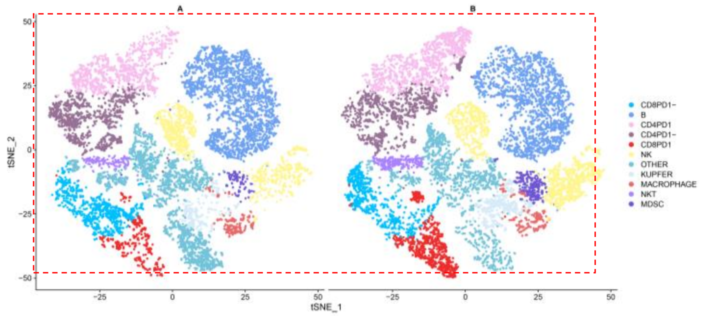

D

		RFP					mEPDR1				
Percentage of cell groups(%)	CD8PD1-	0.079	0.152	0.078	0.067	0.074	0.087	0.068	0.063	0.092	0.115
	B	0.146	0.245	0.233	0.226	0.279	0.164	0.237	0.141	0.268	0.248
	CD4PD1	0.144	0.093	0.099	0.064	0.111	0.140	0.125	0.168	0.075	0.161
	CD4PD1-	0.125	0.135	0.104	0.112	0.122	0.094	0.092	0.114	0.098	0.099
	CD8PD1	0.075	0.027	0.024	0.031	0.025	0.127	0.082	0.093	0.074	0.055
	NK	0.173	0.091	0.124	0.116	0.108	0.102	0.128	0.152	0.148	0.096
	KUPFER	0.030	0.022	0.036	0.044	0.020	0.111	0.044	0.047	0.044	0.016
	MACROPHAGE	0.021	0.025	0.014	0.044	0.027	0.013	0.029	0.023	0.020	0.019
	NKT	0.036	0.015	0.013	0.025	0.014	0.024	0.027	0.025	0.038	0.012
	MDSC	0.021	0.007	0.012	0.011	0.012	0.024	0.032	0.048	0.030	0.011

E

PD1		TIM3		IFN		GzmB		
RFP	mEPDR1	RFP	mEPDR1	RFP	mEPDR1	RFP	mEPDR1	% of CD8+ T cells
24.4	51.1	6.47	25.4	41	12.7	13	10.8	
31.4	57.3	6.94	12.4	39	15.8	13.8	12.3	
31.1	52.7	5.49	25.8	37.8	14.3	27	11.4	
46.5	51	8.09	24.7	42.3	12.6	13.9	7.78	
34.1	53.5	7.45	24.6	27.8	16	18.8	17.6	
44.8	58	7.86	13.2	33.9	11.8	29.2	8.31	

G

	PD1		TIM3		IFN		GzmB	
% of CD8+ T cells	EV	EPDR1	EV	EPDR1	EV	EPDR1	EV	EPDR1
	19.3	28.8	14.2	37.6	20.6	11.4	33.5	6.67
	19.5	33.6	6.6	39.1	20	9.7	37.9	7.46
	24.1	28	20.6	37.5	22.8	16	29.1	15.3

H

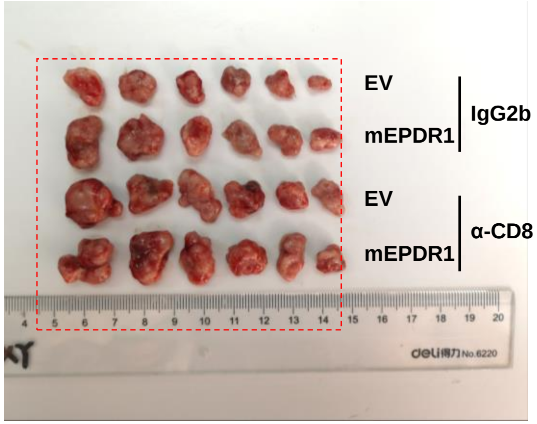

I

	Days	EV + IgG2b						mEPDR1 + IgG2b						EV + α-CD8						mEPDR1 + α-CD8					
Tumor volume	10	176.6	93.9	105.7	99.8	48.4	50.6	171.7	207.4	160.9	86.7	106.4	80.3	103.9	60.9	91.1	46.3	50.7	60.5	261.6	176.4	132.1	130.4	79.5	38.3
	13	205.4	164.5	126.0	100.0	57.5	39.8	417.7	546.4	282.9	184.2	215.5	165.4	329.0	200.5	182.8	149.7	131.8	125.1	425.9	351.2	275.5	363.1	139.3	116.3
	16	455.7	343.5	320.4	323.0	150.3	72.9	728.4	817.5	435.4	267.3	247.4	225.6	616.4	570.8	614.4	554.3	266.3	353.9	817.3	515.7	411.0	456.5	313.6	208.3
	19	615.8	504.8	367.8	397.2	235.2	94.9	966.5	1097.1	664.6	455.8	291.1	294.2	1083.4	844.9	952.6	1176.6	395.6	531.9	1357.6	1260.9	1077.2	759.0	619.8	402.7
	22	697.2	578.5	426.2	423.8	266.4	173.4	1254.3	1261.1	945.1	697.6	552.7	505.1	2146.1	1317.1	1277.0	1307.5	700.0	650.8	2252.6	1816.2	1279.2	1177.3	1041.7	783.2

J

Tumor weight	EV + IgG2b						mEPDR1 + IgG2b						EV + α-CD8						mEPDR1 + α-CD8					
	0.39	0.21	0.24	0.42	0.15	0.12	0.624	0.832	0.432	0.304	0.56	0.592	0.648	1.152	0.558	0.432	0.774	0.792	0.864	0.684	0.9	1.044	0.648	0.72
