# Supplementary material for: EPDR1 promotes PD-L1 expression and tumor immune evasion by inhibiting TRIM21-dependent ubiquitylation of IkappaB kinase-β
Source: EMBO J. 2024 Aug 16;43(19):4248–73. doi: 10.1038/s44318-024-00201-6 (PMC11445549; doi:10.1038/s44318-024-00201-6)
Supplement: Supplementary file 5 — Source data Fig. 3 [file 44318_2024_201_MOESM5_ESM.zip › EMBOJ-2023-116324_SourceDataForFigure3A-H_J-O.pdf]

A

	NTC			shEPDR1-1			shEPDR1-2			
PDL1	1.433955	0.870551	0.80107	0.510506	0.309927	0.447513	0.389582	0.181747	0.255253	Relative mRNA level
B7-1	1.028114	1	0.97942	0.726986	0.697372	0.678302	0.90125	0.926588	1.013959	
B7-H2	0.97942	1	1.028114	0.876606	1.042466	0.986233	0.779165	1.248331	1.356604	
B7-H3	1.057018	0.840896	1.117287	0.858565	0.965936	1.021012	0.779165	0.835088	0.806642	
B7-H4	1.06437	1.337928	0.697372	0.363493	0.293209	0.503478	0.806642	0.692555	0.702222	
PDL2	0.939523	0.90125	1.172835	0.624165	0.646176	0.408951	0.790041	0.870551	0.692555	
GL-9	0.986233	1.035265	0.97942	0.702222	0.920188	0.687771	1.035265	0.952638	1.06437	
IRF1	1.094294	0.97942	0.933033	0.920188	0.876606	0.90125	0.528509	0.624165	0.628507	
IRF2	1.101905	0.926588	0.97942	0.539614	0.732043	0.619854	0.721965	0.602904	0.732043	
VISTA	0.972655	1.057018	0.972655	1.214195	1.125058	0.952638	1.347234	1.36604	1.484524	
HVEM	0.965936	1	1.042466	0.870551	0.97942	1.028114	1.013959	1.086735	1.248331	
EPCAM	0.946058	1.049717	1	0.692555	0.664343	0.63728	0.812252	0.692555	0.835088	
GPC3	1.125058	0.97942	0.913831	1.109569	1.156688	1.125058	0.757858	0.716978	0.747425	
CD70	0.90125	1.356604	0.817902	1.257013	2.329467	1.265757	0.737135	0.473029	1.189207	
OX40L	0.920188	0.840896	1.301342	1.180993	0.952638	1.42405	1.006956	0.716978	1.189207	
CD226	1.189207	0.747425	1.125058	2.20381	0.790041	1.94531	0.463294	0.840896	1.049717	
4-1BBL	1	1.035265	0.965936	1.248331	1.337928	1.197479	0.752623	0.712025	0.80107	
HLA-B	1.042466	0.933033	1.035265	1.006956	0.959264	0.920188	0.521233	0.550953	0.628507	
CEA4	0.965936	0.90125	1.156688	0.752623	0.732043	0.590496	0.870551	0.972655	0.82932	
CEA18	1.086735	0.858565	1.079228	1.283426	1.328686	1.22264	0.668964	0.668964	0.632878	
PVR	0.920188	1.140764	0.946058	1.094294	0.570382	0.732043	1.164734	1.094294	1.006956	
IDO	1.006956	1.013959	0.97942	0.993092	0.790041	0.913831	0.628507	0.582367	0.528509	
HLA-A	0.986233	0.933033	1.094294	0.864537	0.737135	0.678302	0.823591	0.882703	0.913831	

B

	EV	EPDR1
MFI of PE PD-L1	3427	5299
	3528	4834
	3102	4627

C

	NTC	shEPDR1-1	shEPDR1-2
MFI of PE PD-L1	3471	2049	2137
	3544	2153	2181
	2783	2156	2190

C

Pathways	Count	PValue	change
Malaria	8	6.41E-05	UP
cAMP signaling pathway	12	0.006504	UP
African trypanosomiasis	5	0.006649	UP
Ras signaling pathway	12	0.009165	UP
Leukocyte transendothelial migration	8	0.009249	UP
Pathways in cancer	27	1.07E-05	DOWN
Calcium signaling pathway	16	1.16E-04	DOWN
Melanogenesis	9	7.54E-04	DOWN
NF-kappa B signaling pathway	9	9.15E-04	DOWN
Th1 and Th2 cell differentiation	8	0.002033	DOWN

E

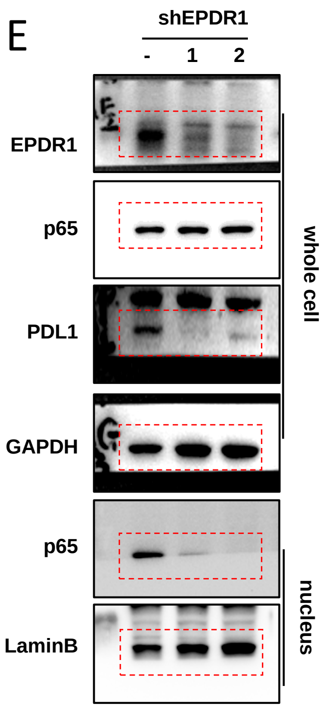

F

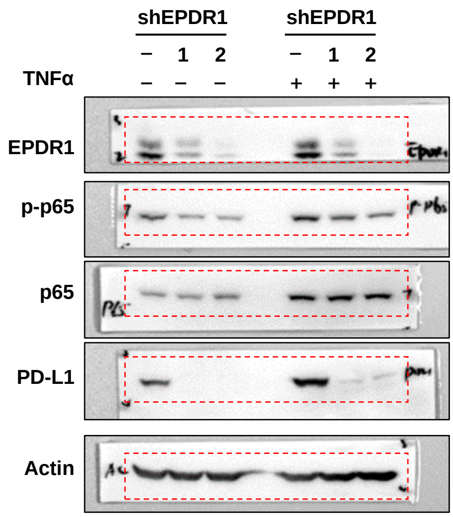

G

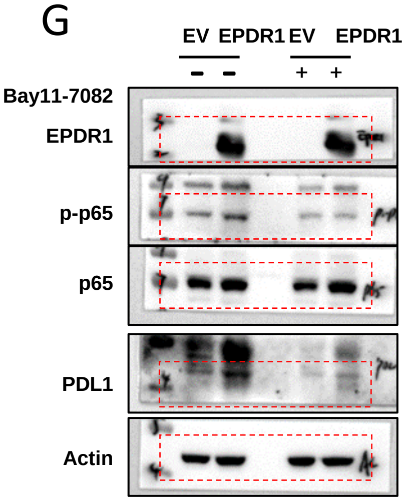

H

		NTC	shEPDR1
Relative mRNA level	IgG	0.053432	0.039661
		0.024245	0.024584
		0.024414	0.032439
	Flag-p65	0.235509	0.086801
		0.138107	0.031552
		0.250669	0.060954

J

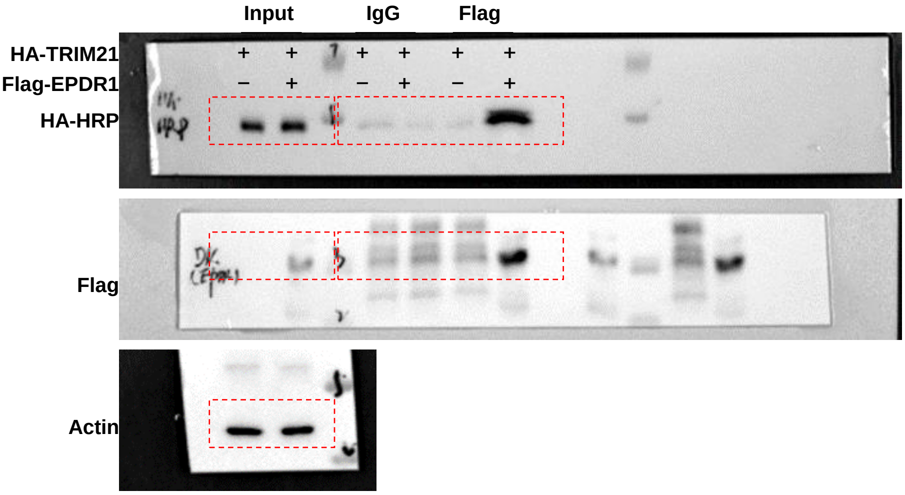

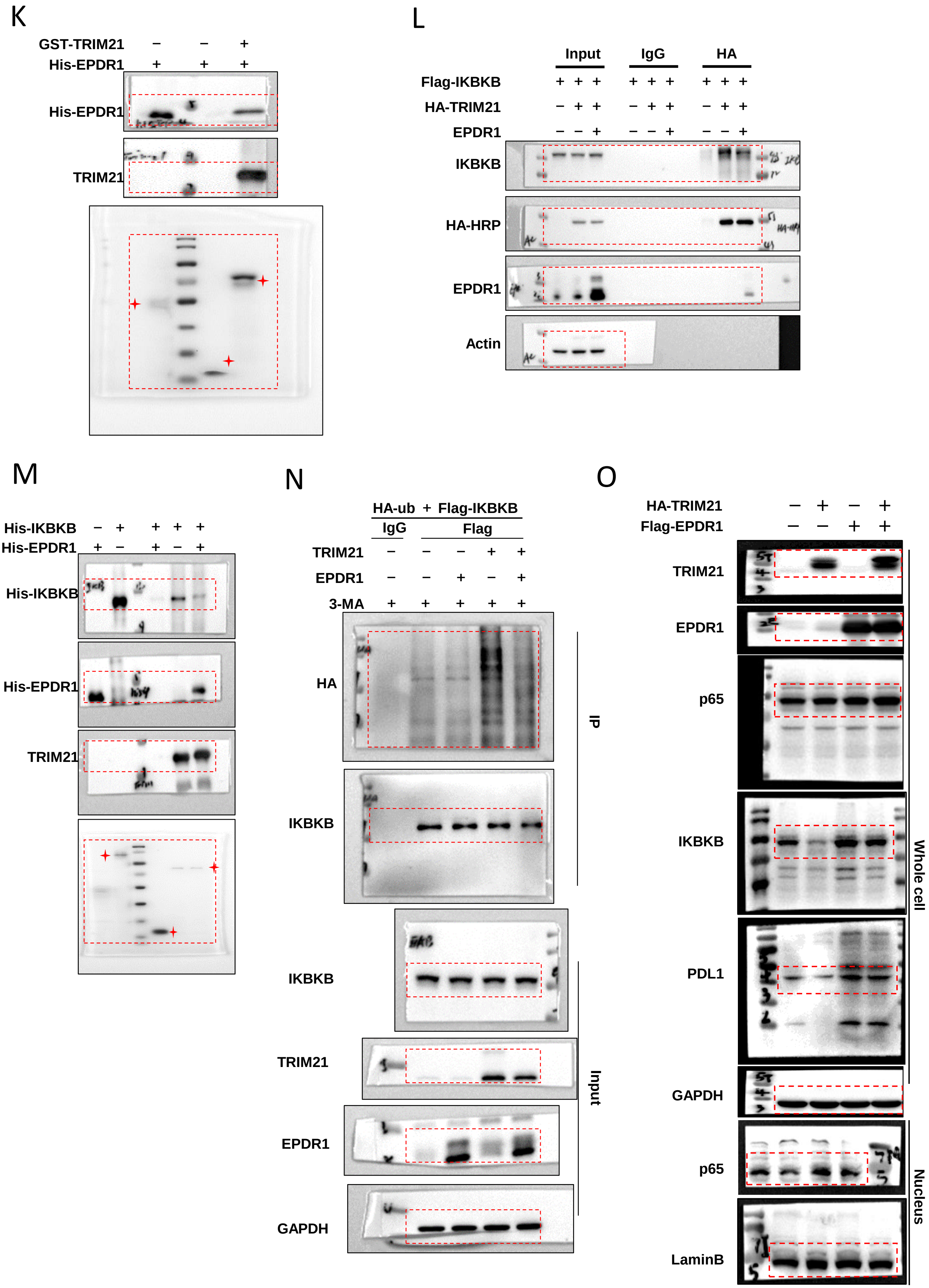
K
GST-TRIM21 − − +
His-EPDR1 + + +
His-EPDR1
TRIM21
L
Input
IgG
HA
Flag-IKBKB + + + + + + + + +
HA-TRIM21 − + + − + + − + +
EPDR1 − − + − − + − − +
IKBKB
HA-HRP
EPDR1
Actin
M
His-IKBKB − + + + +
His-EPDR1 + − + − +
His-IKBKB
His-EPDR1
TRIM21
N
HA-ub + Flag-IKBKB
IgG
Flag
TRIM21 − − − + +
EPDR1 − − + − +
3-MA + + + + +
HA
IKBKB
IP
IKBKB
TRIM21
EPDR1
GAPDH
Input
O
HA-TRIM21 − + − +
Flag-EPDR1 − − + +
TRIM21
EPDR1
p65
IKBKB
PDL1
GAPDH
Whole cell
p65
LaminB
Nucleus
