# Supplementary material for: EPDR1 promotes PD-L1 expression and tumor immune evasion by inhibiting TRIM21-dependent ubiquitylation of IkappaB kinase-β
Source: EMBO J. 2024 Aug 16;43(19):4248–73. doi: 10.1038/s44318-024-00201-6 (PMC11445549; doi:10.1038/s44318-024-00201-6)
Supplement: Supplementary file 6 — Source data Fig. 4 [file 44318_2024_201_MOESM6_ESM.pdf]

A

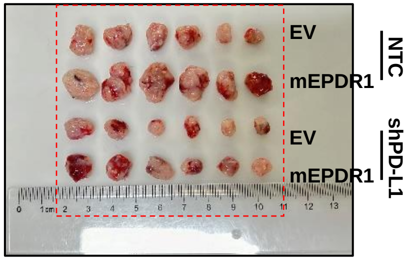

B

	Days	EV + NTC						mEPDR1 + NTC						EV + shEPDR1						mEPDR1 + shEPDR1					
Tumor Volume (mm^3)	10	75.1	78.8	93.5	82.6	29.7	27.1	130.9	113.1	134.2	99.2	64.8	39.0	120.7	133.0	61.9	65.5	35.2	31.0	106.6	124.8	101.5	55.1	29.6	37.4
	13	240.5	172.3	162.1	127.3	85.3	36.6	264.0	168.0	225.4	141.0	102.9	68.4	151.8	152.9	69.9	82.7	37.2	33.9	190.3	204.7	177.1	69.3	36.8	93.3
	16	310.9	265.2	200.6	168.4	138.3	53.1	500.0	475.7	454.8	407.4	169.6	165.3	189.2	239.0	79.8	118.3	46.9	52.7	438.0	325.4	279.7	137.5	126.1	113.3
	19	496.9	416.9	291.9	251.3	156.8	72.9	708.0	722.5	629.1	597.0	240.2	399.4	417.7	319.5	97.8	170.7	59.1	77.5	576.6	435.1	309.5	188.9	206.4	156.6
	22	488.4	645.5	432.1	538.2	150.2	147.9	1096.3	981.6	1169.1	969.4	394.9	450.7	523.8	385.7	142.7	193.8	68.5	128.5	831.2	523.5	386.9	222.8	249.1	213.6

C

Tumor weight (g)	EV + NTC						mEPDR1 + NTC						EV + shEPDR1						mEPDR1 + shEPDR1					
	0.58	0.4	0.41	0.33	0.29	0.37	0.82	0.9	1	0.71	0.48	0.42	0.3	0.18	0.15	0.12	0.11	0.08	0.42	0.53	0.2	0.39	0.34	0.2

D-E

% of $CD8^+$ T cells		EV + NTC						mEPDR1 + NTC						EV + shEPDR1						mEPDR1 + shEPDR1					
	PD1	52.1	52.8	49.6	51.9	56.3	47.5	77.2	63.2	61.9	60	59.8	59.7	22	21.8	9.68	14	16.9	17.6	16	24.6	19.4	27.1	22.9	19.6
	TIM3	42.7	41.5	54.9	24.8	25.9	28.1	80.7	57.9	52.4	51.6	55.4	41.8	7.32	12.1	10.8	16.3	11.6	5.51	24.4	13.6	10.8	16.7	22.9	24.3
	IFNγ	25.5	24.6	27.2	22.7	25.6	21.8	9.65	15.8	20.2	10.7	8.97	16.8	44.8	35	35.4	43.7	32.8	47.3	48.1	32	44.9	49.1	43	37.1
	GZMB	25.3	29.9	27.9	21.1	26.5	23.2	16.6	21.3	22.1	18.6	16.8	23.9	36.8	37.5	37.4	36	35.2	37.9	34.2	36.1	34.6	35.4	30.1	31.2

F

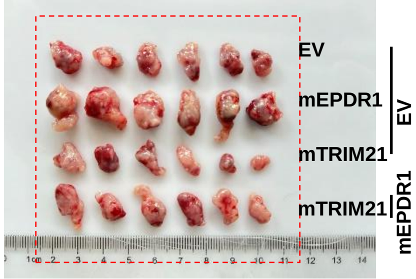

G

	Days	EV + EV						mEPDR1 + EV						EV + mTRIM21						mEPDR1 + mTRIM21					
Tumor Volume (mm^3)	10	125.6	98.3	86.1	113.2	70.8	45.6	134.7	122.8	128.3	78.7	66.8	72.8	38.2	58.3	48.8	27.2	25.6	22.0	69.1	57.9	44.2	58.1	32.8	43.7
	13	200.9	137.1	161.9	110.7	121.7	94.2	251.0	222.1	216.8	164.0	140.7	132.4	73.0	100.1	103.2	42.3	20.0	14.2	194.4	134.9	107.6	92.4	79.4	66.0
	16	308.9	186.3	194.8	160.1	177.6	129.6	422.2	418.7	322.7	255.1	280.4	288.0	186.7	160.5	130.1	72.3	41.7	23.1	348.0	182.0	190.0	158.0	131.1	121.6
	19	503.0	200.4	347.1	263.3	252.3	252.8	678.0	773.3	575.8	470.7	589.7	425.5	295.7	279.0	127.6	103.3	89.3	77.9	508.6	258.2	304.9	278.9	224.7	183.9
	22	692.4	297.3	440.7	391.8	390.6	314.7	999.9	1312.3	947.9	666.5	834.3	734.7	409.0	369.8	234.0	167.9	174.7	120.6	660.2	416.8	626.4	502.6	423.7	288.2

H

	EV + EV						mEPDR1 + EV						EV + mTRIM21						mEPDR1 + mTRIM21					
Tumor weight (g)	0.52	0.42	0.38	0.31	0.3	0.25	0.81	0.9	0.77	0.59	0.55	0.48	0.24	0.24	0.23	0.17	0.15	0.1	0.45	0.36	0.41	0.36	0.32	0.25

I

	EV + EV						mEPDR1 + EV						EV + mTRIM21						mEPDR1 + mTRIM21					
mumbrane-bound PDL1	1299	1145	1064	1080	1161	815	2083	1983	2398	2851	2518	3039	407	606	590	606	710	782	750	1511	1667	1552	1495	1626

J-K

		EV + EV						mEPDR1 + EV						EV + mTRIM21						mEPDR1 + mTRIM21					
% of $CD8^+$ T cells	PD1	54.7	57.6	65.2	65.9	64.9	65.3	79.5	84.4	82.8	81.4	80.6	82.2	30.2	29.7	49.5	50.4	50.3	51.1	66.7	69.3	64.9	62.7	70	65.9
	TIM3	40.1	43.4	46.2	45.2	44.4	52.7	60.7	59.1	66.8	67.9	57.6	59.3	21.6	21.3	38.2	38.4	30.3	32.2	48.2	45.3	41.9	46.6	56.9	54.6
	IFNγ	34.4	37.7	34.6	42.1	41.5	40.4	28.2	28	16.6	29.6	29.9	17	62.5	61.1	52.7	58.7	62.6	57.8	46.6	38.1	48.3	60	46.8	35.3
	GZMB	32.5	25.3	30.1	30.1	30.2	33.4	23.1	32.6	20.5	22.5	25.9	20.6	40.7	40.7	42.1	40.2	36.3	41.8	34.5	33.4	36.1	36.9	37.2	32.7
