# Supplementary figures and images for: EPDR1 promotes PD-L1 expression and tumor immune evasion by inhibiting TRIM21-dependent ubiquitylation of IkappaB kinase-β
Source: EMBO J. 2024 Aug 16;43(19):4248–73. doi: 10.1038/s44318-024-00201-6 (PMC11445549; doi:10.1038/s44318-024-00201-6)

Low-EPDR1

#1

#2

High-EPDR1

# 1

# 2

EPDR1

PD-L1

P65

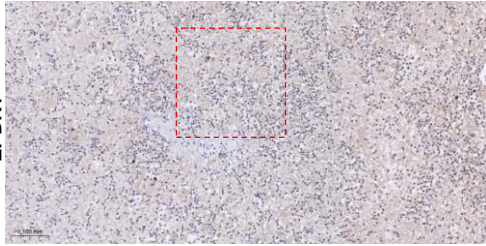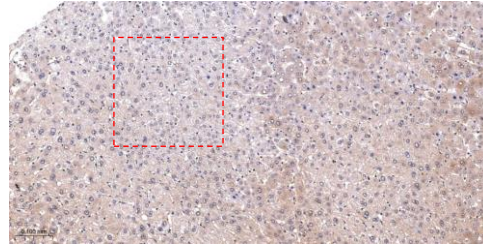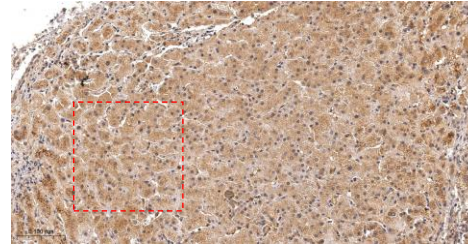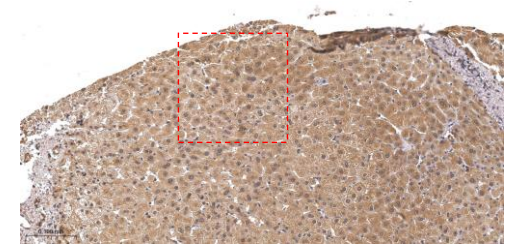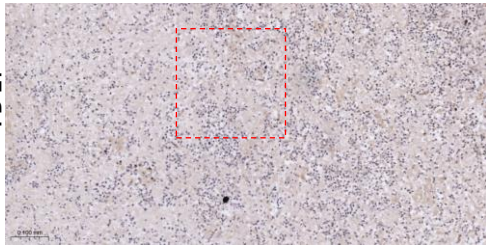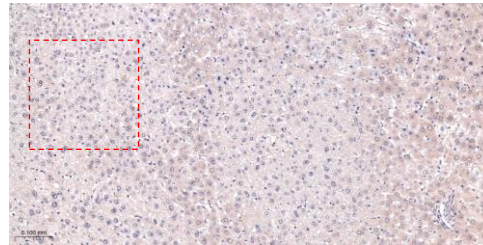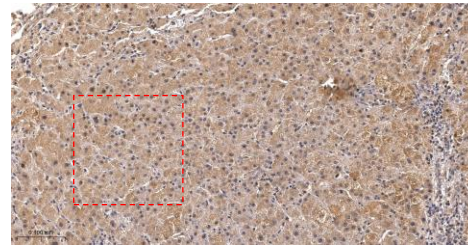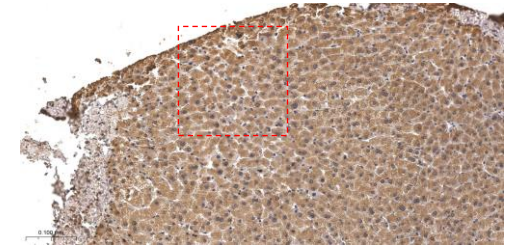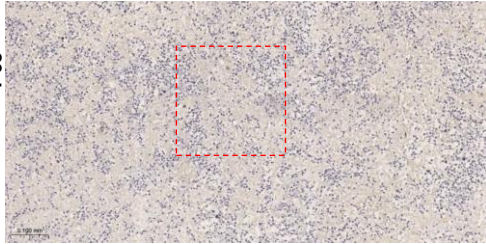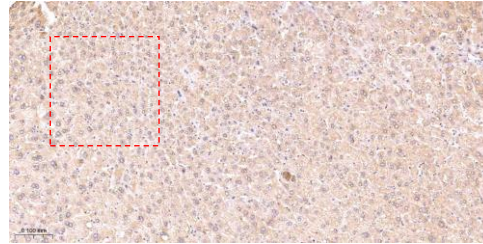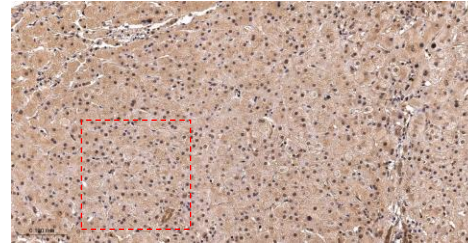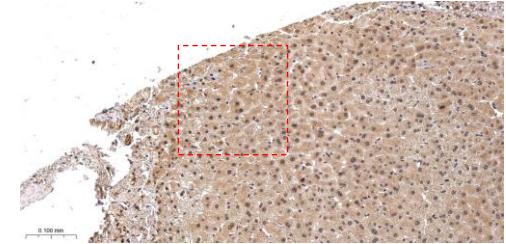

B

| Signal intensity |          |
|------------------|----------|
| EPDR1            | PD-L1    |
| 23.978           | 23.9269  |
| 23.64513         | 23.70632 |
| 24.79324         | 25.09748 |
| 25.14617         | 23.49624 |
| 22.85053         | 23.67405 |
| 24.97447         | 25.06713 |
| 23.28599         | 23.30574 |
| 23.73137         | 23.78069 |
| 25.57482         | 24.60778 |
| 25.70035         | 25.93228 |
| 26.24613         | 24.83994 |
| 26.31447         | 26.19659 |
| 25.55958         | 24.73905 |
| 24.9487          | 23.54139 |
| 26.09572         | 25.75469 |
| 25.65387         | 25.50937 |
| 24.57896         | 23.70148 |
| 23.44595         | 23.6154  |
| 26.41379         | 26.06084 |
| 25.6471          | 25.53433 |
| 25.69147         | 26.21824 |
| 25.31567         | 25.93513 |
| 25.76404         | 25.4862  |
| 23.69124         | 24.03556 |
| 26.03237         | 25.53144 |
| 26.25054         | 25.10389 |
| 26.55269         | 26.45456 |
| 26.42729         | 24.19757 |
| 24.45955         | 23.67289 |
| 26.20003         | 25.62985 |
| 26.04399         | 26.02247 |
| 24.90934         | 24.12653 |
| 25.51214         | 25.88843 |
| 25.41            | 23.79717 |
| 23.80627         | 24.46458 |
| 24.88936         | 24.42269 |
| 26.25639         | 23.64842 |
| 26.24872         | 26.48864 |
| 24.2451          | 22.82433 |
| 24.53968         | 23.91339 |
| 25.02934         | 25.11748 |
| 25.37948         | 25.59431 |
| 24.16233         | 22.58782 |
| 24.59115         | 24.99377 |
| 24.36859         | 24.19778 |
| 23.94137         | 23.4503  |
| 24.81715         | 25.3031  |
| 24.57252         | 24.30908 |
| 24.75195         | 23.91438 |
| 23.65488         | 24.23541 |

C

| Signal intensity |           |
|------------------|-----------|
| EPDR1            | Nucl. p65 |
| 23.978           | 21.20295  |
| 23.64513         | 18.99158  |
| 24.79324         | 19.8565   |
| 25.14617         | 20.62167  |
| 22.85053         | 18.91934  |
| 24.97447         | 20.6096   |
| 23.28599         | 20.23275  |
| 23.73137         | 21.68463  |
| 25.57482         | 22.73596  |
| 25.70035         | 23.15468  |
| 26.24613         | 21.96197  |
| 26.31447         | 21.17595  |
| 25.55958         | 22.85623  |
| 24.9487          | 20.74934  |
| 26.09572         | 22.8551   |
| 25.65387         | 22.63565  |
| 24.57896         | 22.72268  |
| 23.44595         | 18.89397  |
| 26.41379         | 23.00444  |
| 25.6471          | 22.98126  |
| 25.69147         | 22.44151  |
| 25.31567         | 22.3452   |
| 25.76404         | 22.31599  |
| 23.69124         | 21.74724  |
| 26.03237         | 22.81682  |
| 26.25054         | 23.38209  |
| 26.55269         | 23.51102  |
| 26.42729         | 22.37535  |
| 24.45955         | 20.41451  |
| 26.20003         | 22.90062  |
| 26.04399         | 22.82124  |
| 24.90934         | 22.42533  |
| 25.51214         | 22.54498  |
| 25.41            | 22.68248  |
| 23.80627         | 21.31041  |
| 24.88936         | 21.95604  |
| 26.25639         | 21.26621  |
| 26.24872         | 23.00217  |
| 24.2451          | 19.00854  |
| 24.53968         | 21.5078   |
| 25.02934         | 22.56384  |
| 25.37948         | 22.78239  |
| 24.16233         | 21.37332  |
| 24.59115         | 22.00527  |
| 24.36859         | 20.48535  |
| 23.94137         | 21.11791  |
| 24.81715         | 21.23109  |
| 24.57252         | 22.55307  |
| 24.75195         | 22.65172  |
| 23.65488         | 20.57166  |

Supplement: Supplementary file 7 — Source data Fig. 5 [file 44318_2024_201_MOESM7_ESM.pdf]

A

|     | EV       |          |          | EPDR1    |          |          | PDL1 mRNA stability<br>(fold change over 0 h) |
|-----|----------|----------|----------|----------|----------|----------|-----------------------------------------------|
| 0h  | 1        | 1        | 1        | 1        | 1        | 1        |                                               |
| 5h  | 0.269807 | 0.386891 | 0.243164 | 0.334482 | 0.234881 | 0.543367 |                                               |
| 10h | 0.1895   | 0.1539   | 0.1696   | 0.2365   | 0.25     | 0.2952   |                                               |

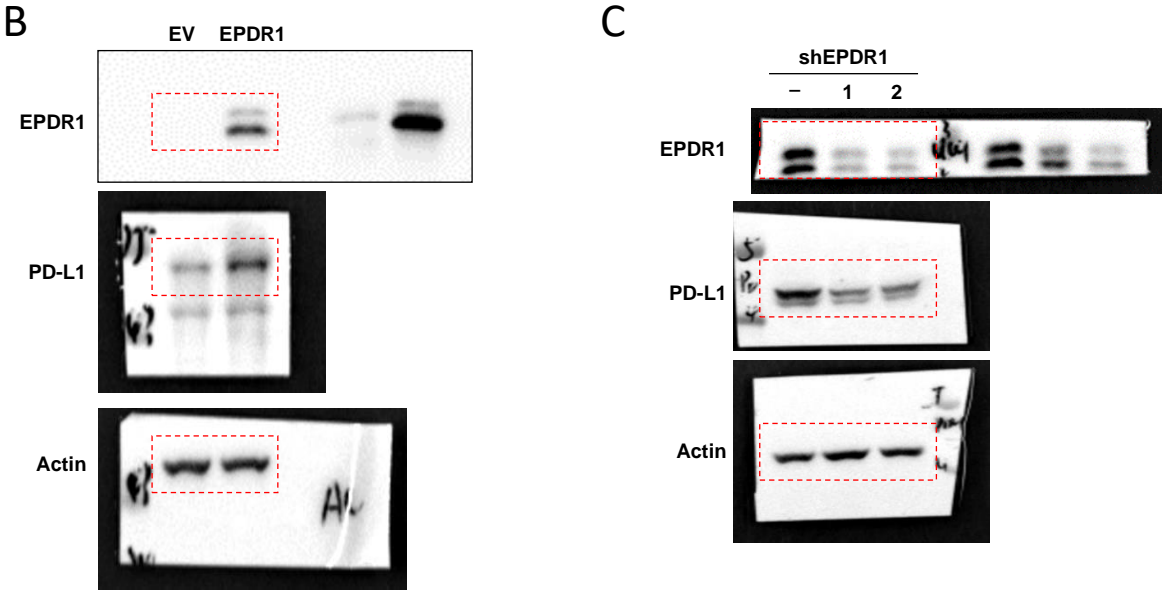

D

|                 | NTC  | shEPDR1-1 | shEPDR1-2 |
|-----------------|------|-----------|-----------|
| MFI of PE PD-L1 | 1700 | 694       | 1016      |
|                 | 1121 | 726       | 1072      |
|                 | 1478 | 807       | 1113      |
|                 | 1421 | 1032      | 1008      |
|                 | 1397 | 983       | 823       |
|                 | 1446 | 1088      | 1056      |

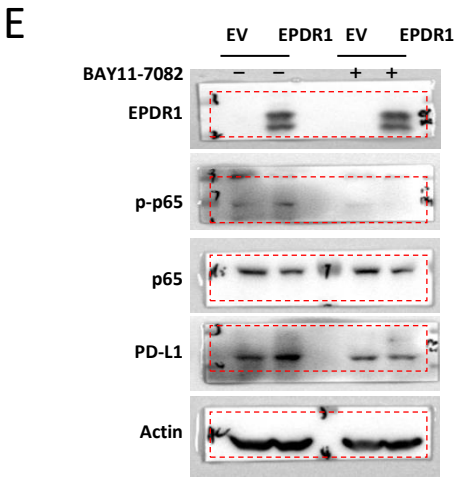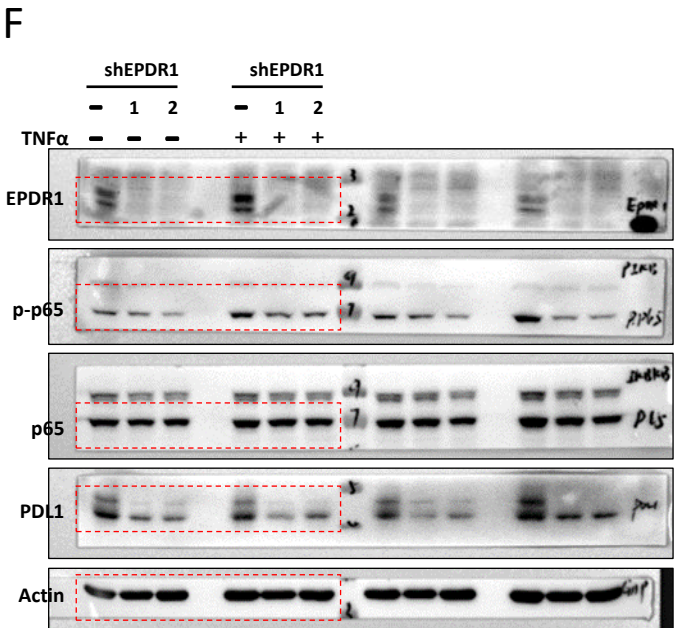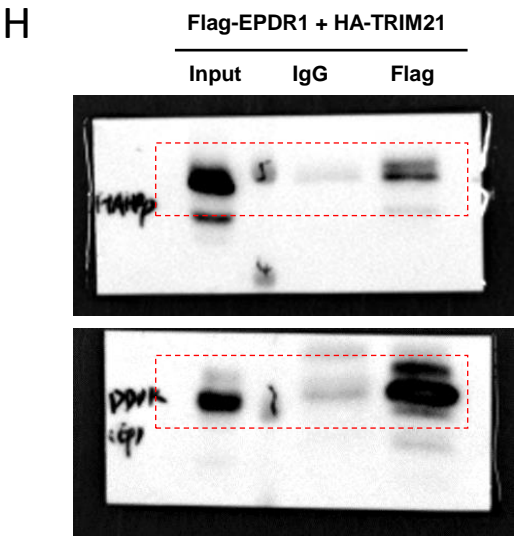

I

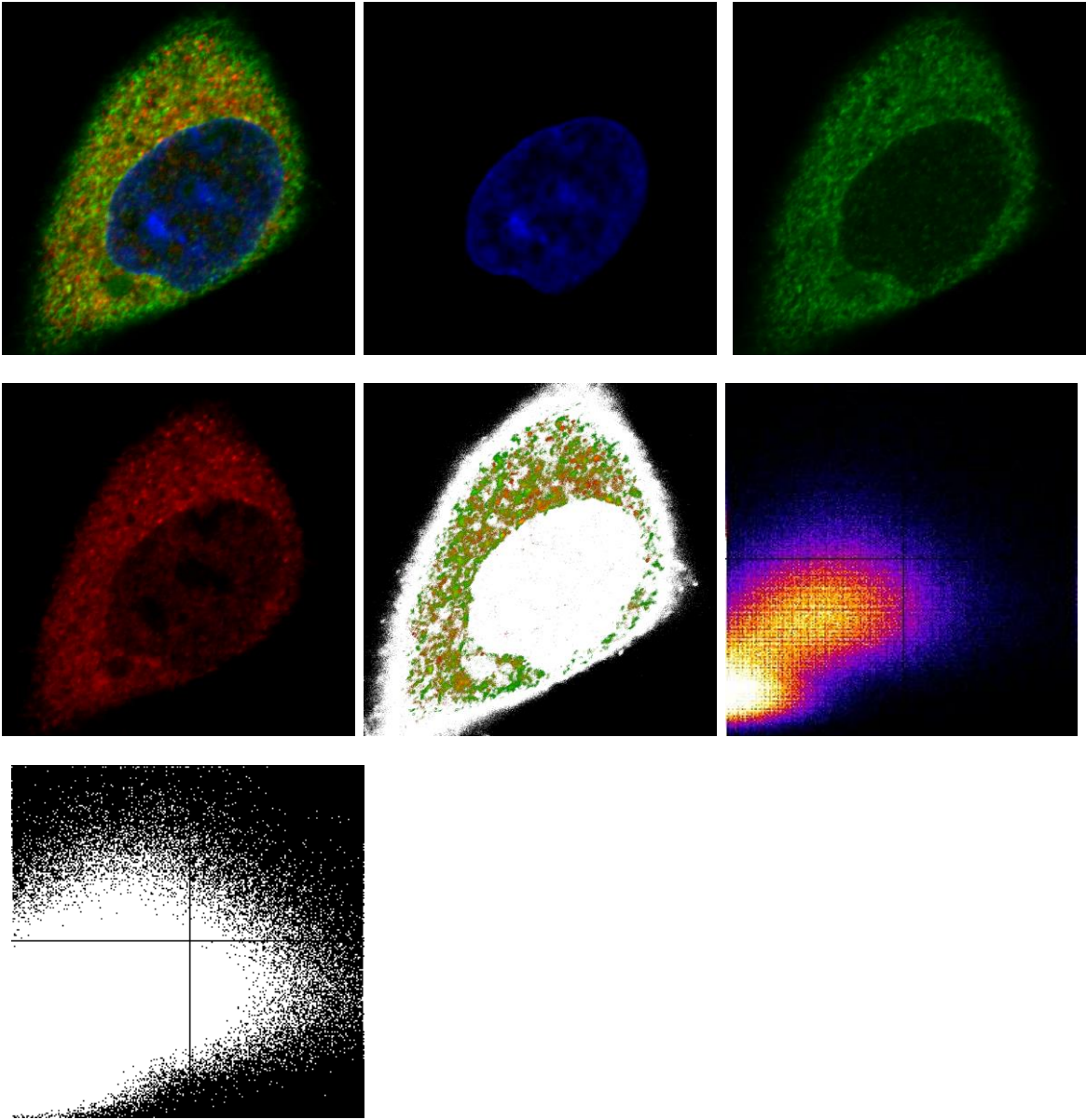

J

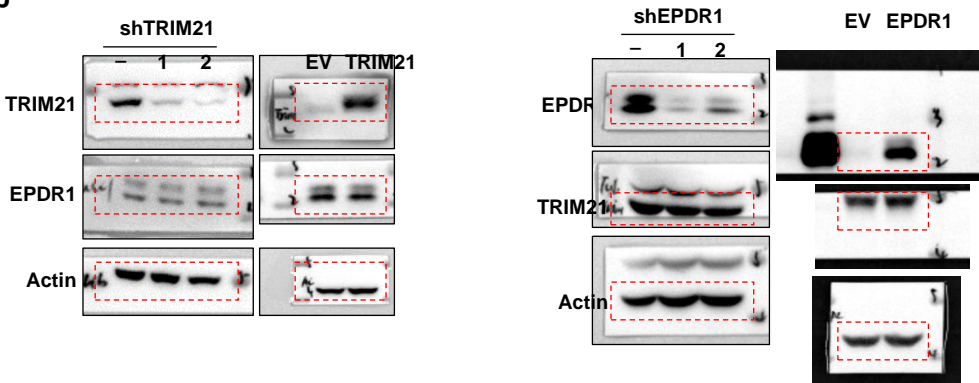

K

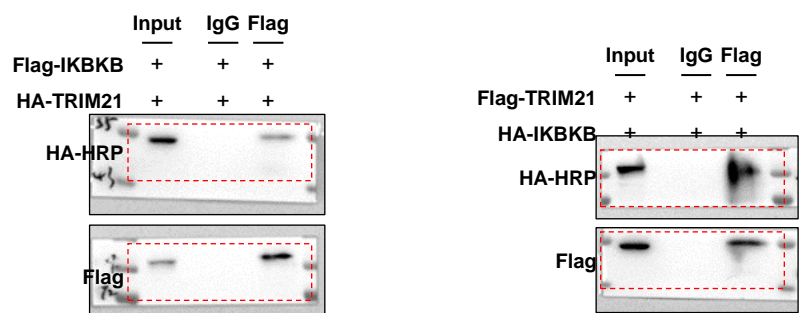

L

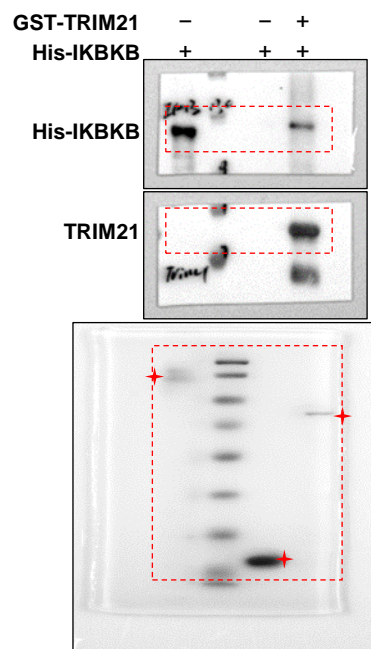

M

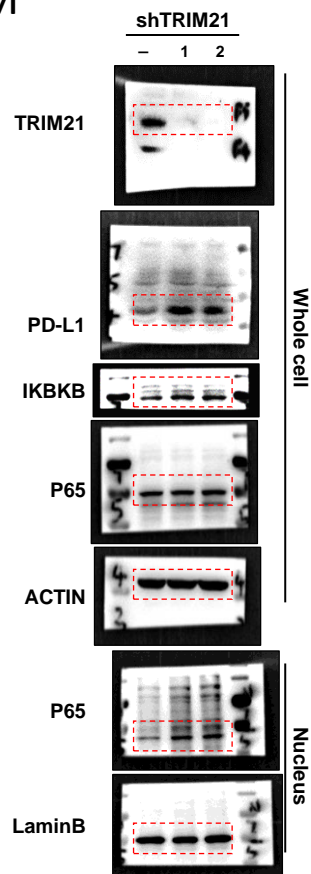

Supplement: Supplementary file 8 — Source Data For Expanded View Figures and Appendix Figures [file 44318_2024_201_MOESM8_ESM.zip › EMBOJ-2023-116324_SourceDataForExpandedView/EMBOJ-2023-116324_SourceDataForExpanded View Figure 3.pdf]
