# Supplementary material for: EPDR1 promotes PD-L1 expression and tumor immune evasion by inhibiting TRIM21-dependent ubiquitylation of IkappaB kinase-β
Source: EMBO J. 2024 Aug 16;43(19):4248–73. doi: 10.1038/s44318-024-00201-6 (PMC11445549; doi:10.1038/s44318-024-00201-6)
Supplement: Supplementary file 8 — Source Data For Expanded View Figures and Appendix Figures [file 44318_2024_201_MOESM8_ESM.zip › EMBOJ-2023-116324_SourceDataForExpandedView/EMBOJ-2023-116324_SourceDataForAppendix Figure S1.pdf]

A

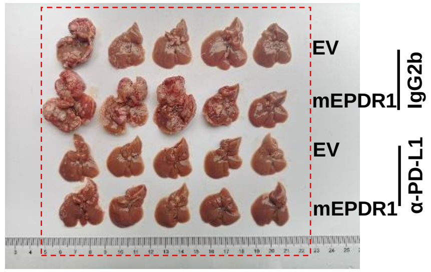

B

No. Of tumor nodules	EV + IgG2b					mEPDR1 + IgG2B					EV + α-PD-L1					mEPDR1 + α-PD-L1				
	28	22	10	9	12	24	31	42	38	19	9	10	6	7	4	11	8	12	3	5

C, D

% of CD8⁺ T cells		EV + IgG2b					mEPDR1 + IgG2B					EV + α-PD-L1					mEPDR1 + α-PD-L1				
	PD1	48.9	48.8	59.9	47	55.4	70.5	68.1	61.4	59.2	83.4	21.9	23.4	22.1	19.8	31.1	32.6	23.9	23.2	38.1	24.2
	TIM3	17.5	19.3	15.1	24.7	16	30.9	29	24.9	23.6	22.8	3.16	2.59	5.74	2.23	7.19	7.19	7.81	6.95	9.5	6.13
	IFN	33.2	25.2	37.7	45	28.2	25.2	26.4	23.7	20.9	16.3	39.9	51.6	49.8	54.6	51.2	42	38.3	37.5	50.5	48
	GZMB	47.3	52.4	41.5	41.9	32.7	23.8	22.5	24.8	25.2	31	48.9	30.9	53.9	48.1	57.7	64.4	50.7	46.7	25.6	43.7
