# Supplementary material for: EPDR1 promotes PD-L1 expression and tumor immune evasion by inhibiting TRIM21-dependent ubiquitylation of IkappaB kinase-β
Source: EMBO J. 2024 Aug 16;43(19):4248–73. doi: 10.1038/s44318-024-00201-6 (PMC11445549; doi:10.1038/s44318-024-00201-6)
Supplement: Supplementary file 8 — Source Data For Expanded View Figures and Appendix Figures [file 44318_2024_201_MOESM8_ESM.zip › EMBOJ-2023-116324_SourceDataForExpandedView/EMBOJ-2023-116324_SourceDataForAppendix Figure S2.pdf]

A

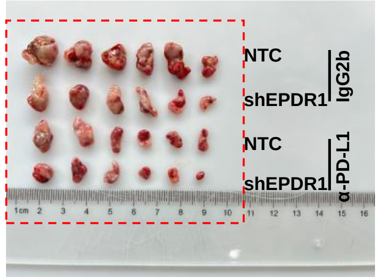

Tumor Volume (mm³) Days	EV + IgG2b						shEPDR1 + IgG2B						EV + α-PD-L1						shEPDR1 + α-PD-L1					
10	160.1	156.8	100.3	63.4	53.5	40.2	80.9	73.5	55.4	62.8	26.2	22.2	31.4	24.6	31.0	25.5	39.9	40.1	87.0	70.0	32.2	37.1	11.1	10.8
13	305.0	235.6	158.8	116.0	89.7	67.9	179.0	131.0	86.1	167.5	72.0	46.3	26.9	44.4	34.4	33.0	32.5	47.9	130.2	111.9	46.8	28.9	28.3	18.5
16	381.8	403.7	252.2	152.3	162.0	109.9	254.5	177.4	100.5	59.7	71.3	30.5	46.1	62.4	57.0	51.0	63.0	76.9	181.3	166.4	58.9	32.1	22.2	19.6
19	848.2	537.5	357.2	183.9	212.0	208.1	347.7	233.2	145.6	103.8	151.2	35.4	54.0	79.6	116.0	65.5	142.0	168.4	253.0	196.8	66.0	30.6	25.9	10.9
22	1300.0	740.0	484.4	308.1	364.7	258.8	547.1	366.1	274.1	167.7	178.0	56.9	103.6	136.4	201.2	102.6	262.6	282.3	322.4	283.1	97.6	59.9	43.8	15.5

Tumor weight (g)	EV + IgG2b						shEPDR1 + IgG2B						EV + α-PD-L1						shEPDR1 + α-PD-L1					
	0.69	0.5	0.43	0.32	0.5	0.12	0.33	0.29	0.26	0.19	0.11	0.05	0.32	0.26	0.11	0.09	0.08	0.08	0.18	0.14	0.09	0.04	0.05	0.02

% of CD8⁺ T cells	EV + IgG2b						shEPDR1 + IgG2B						EV + α-PD-L1						shEPDR1 + α-PD-L1					
PD1	55.3	57.8	54.1	49.3	56.1	48	36.8	36.2	43.9	38.1	39.2	39.2	33.8	33.8	28	35.3	31.4	36	35.5	22.1	23.5	31.2	26.9	29.6
TIM3	55.4	59.8	50.7	44.9	52.1	44.9	33.2	33.1	41.3	45.3	36.3	35.3	31.2	32.9	23.4	30.4	30.1	32.7	32.9	20.5	22.2	29.4	25.2	25
IFN	21.1	17.2	18.7	18.1	21.5	26	36.1	35.9	32.6	36.4	34.8	38.5	52.5	45.2	41.7	42.2	42.9	41.3	45.5	58.3	42.7	44.7	56.1	59.6
GZMB	21.6	19.5	30.5	21.6	30.4	31.7	40.8	32.9	43	31.8	39.1	38.2	58	58.9	63.5	45.3	52.7	58.7	54.7	56.5	64.5	64.6	60.1	61.3
