# Supplementary material for: EPDR1 promotes PD-L1 expression and tumor immune evasion by inhibiting TRIM21-dependent ubiquitylation of IkappaB kinase-β
Source: EMBO J. 2024 Aug 16;43(19):4248–73. doi: 10.1038/s44318-024-00201-6 (PMC11445549; doi:10.1038/s44318-024-00201-6)
Supplement: Supplementary file 8 — Source Data For Expanded View Figures and Appendix Figures [file 44318_2024_201_MOESM8_ESM.zip › EMBOJ-2023-116324_SourceDataForExpandedView/EMBOJ-2023-116324_SourceDataForExpanded View Figure 2.pdf]

A

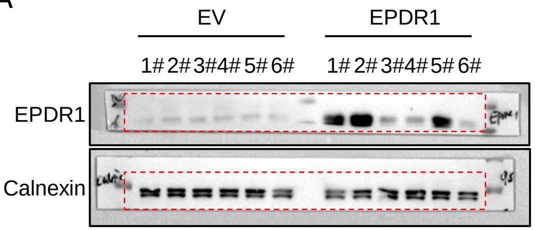

C

% of CD8$^+$ T cells	
PDI+ TIM3+	
RFP	EPDR1
1.55	22
1.96	12.7
0.71	22.6
2.05	22
2.29	21.3
2.21	13.5

E

% of CD8$^+$ T cells		EV			mEPDR1		
	PD1	49.4	50.3	48.5	56.1	51.1	53.8
	TIM3	19.4	17.1	21.6	25.2	23.9	25.7
	IFN	46.8	46.7	47.9	16.7	26.1	18.9
	GZMB	33.4	33.9	30.3	24.3	21.1	23.3

G

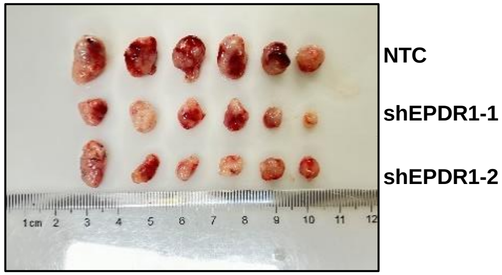

H

	Days	NTC						shEPDR1-1						shEPDR1-2					
Tumor Volume (mm^3)	10	87.8	66.2	55.4	42.5	27.9	15.4	87.5	40.2	36.6	23.1	16.2	12.1	50.9	50.6	30.6	29.2	19.8	14.4
	13	124.2	182.9	159.9	135.9	107.1	38.2	126.7	87.5	63.7	28.9	26.2	20.9	57.6	45.7	33.0	51.7	41.0	24.6
	16	359.2	293.1	213.9	177.4	140.4	68.4	232.5	172.9	108.0	51.1	56.2	50.1	126.4	122.1	98.3	126.5	91.6	45.1
	19	494.0	445.4	338.1	276.5	209.3	85.9	256.6	201.6	140.5	85.6	102.7	51.9	171.3	177.5	166.9	151.1	106.1	49.7
	22	860.5	856.1	480.5	393.6	335.1	194.7	428.8	238.9	211.8	196.5	156.0	73.9	385.9	312.4	152.3	129.4	152.1	90.6

I

		NTC						shEPDR1-1						shEPDR1-2					
Tumor weight (g)		0.56	0.78	0.55	0.53	0.37	0.25	0.26	0.31	0.22	0.26	0.08	0.04	0.42	0.17	0.18	0.19	0.11	0.11

J-K

		NTC						shEPDR1-1						shEPDR1-2					
% of CD8$^+$ T cells	PD1	19.2	25.7	21	25.1	27.4	25.5	15.1	13.9	11.5	10.5	12.5	11.5	14.6	8.23	12.5	7	11.3	12.8
	TIM3	49.9	48	55.6	50.4	60.3	59.1	32.3	33.4	43	34	37.4	37.8	39.9	38.4	45.5	38.9	37.1	49
	IFN	33.5	32.8	41.4	40.6	42.6	38.8	66.5	55.8	60.8	44.8	55.5	52	50.4	45.9	56.8	55.2	44.3	48.8
	GZMB	32.2	33.6	32.9	32.2	46.3	46.3	73	69.1	71.9	46.8	54.9	67.5	54.8	49.9	56	42.2	54.2	53.4
