# Supplementary material for: EPDR1 promotes PD-L1 expression and tumor immune evasion by inhibiting TRIM21-dependent ubiquitylation of IkappaB kinase-β
Source: EMBO J. 2024 Aug 16;43(19):4248–73. doi: 10.1038/s44318-024-00201-6 (PMC11445549; doi:10.1038/s44318-024-00201-6)

A

expression	group
2.017404	normal
1.216301	normal
0.587435	normal
1.174869	normal
0.625248	normal
0.106764	normal
0.467326	normal
10.87166	normal
1.955449	normal
0.318088	normal
0.670124	normal
0.161824	normal
0.535191	normal
2.728532	normal
0.253161	normal
0.200007	normal
0.1014	normal
0.783565	normal
132.7437	Tumor
147.2884	Tumor
18.53911	Tumor
84.59497	Tumor
125.5832	Tumor
65.91341	Tumor
34.35623	Tumor
164.5634	Tumor
3.440218	Tumor
0.60395	Tumor
1.497442	Tumor
4.732164	Tumor
108.9955	Tumor
22.59803	Tumor
11.94326	Tumor
1.131412	Tumor
23.88652	Tumor
138.9214	Tumor

B

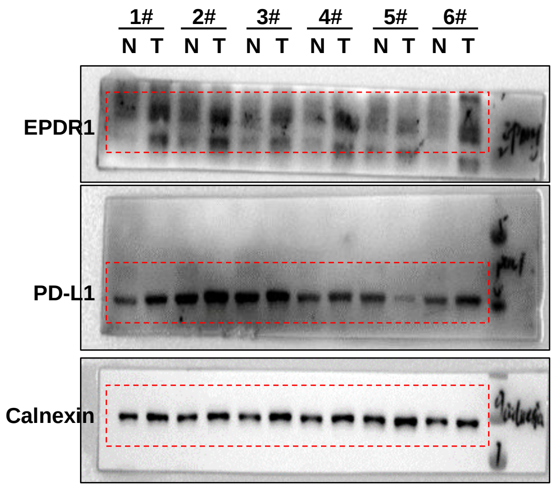

Supplement: Supplementary file 8 — Source Data For Expanded View Figures and Appendix Figures [file 44318_2024_201_MOESM8_ESM.zip › EMBOJ-2023-116324_SourceDataForExpandedView/EMBOJ-2023-116324_SourceDataForExpanded View Figure 5.pdf]
