# Supplementary material for: EPDR1 promotes PD-L1 expression and tumor immune evasion by inhibiting TRIM21-dependent ubiquitylation of IkappaB kinase-β
Source: EMBO J. 2024 Aug 16;43(19):4248–73. doi: 10.1038/s44318-024-00201-6 (PMC11445549; doi:10.1038/s44318-024-00201-6)
Supplement: Supplementary file 9 — Expanded View Figures [file 44318_2024_201_MOESM9_ESM.pdf]

Expanded View Figures

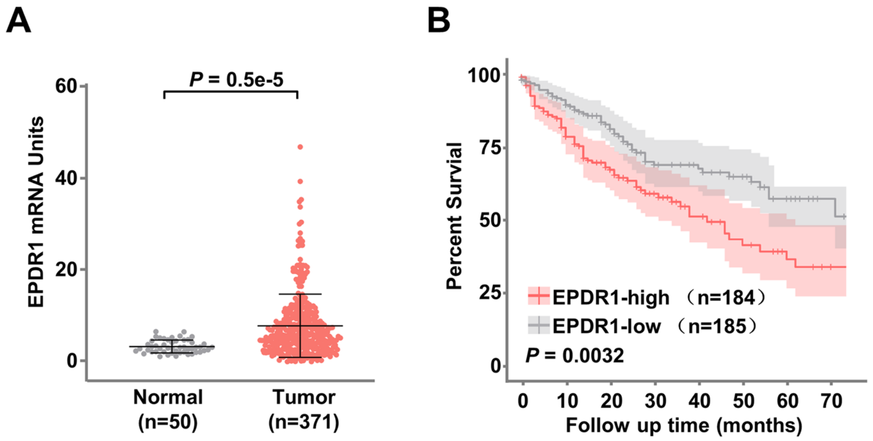

Figure EV1. Aberrant expression of EPDR1 in HCC is associated with immunosuppression.

(**A**) The mRNA levels of EPDR1 were determined in adjacent noncancerous liver tissues (Normal) and liver cancer tissues (Tumor) from The Cancer Genome Atlas (TCGA) database, statistical analyses were performed by two-tailed unpaired Student's *t*-test. (**B**) Kaplan–Meier analysis of overall survival with log-rank tests for HCC patients with low versus high expression of EPDR1. Source data are available online for this figure.

© The Author(s)

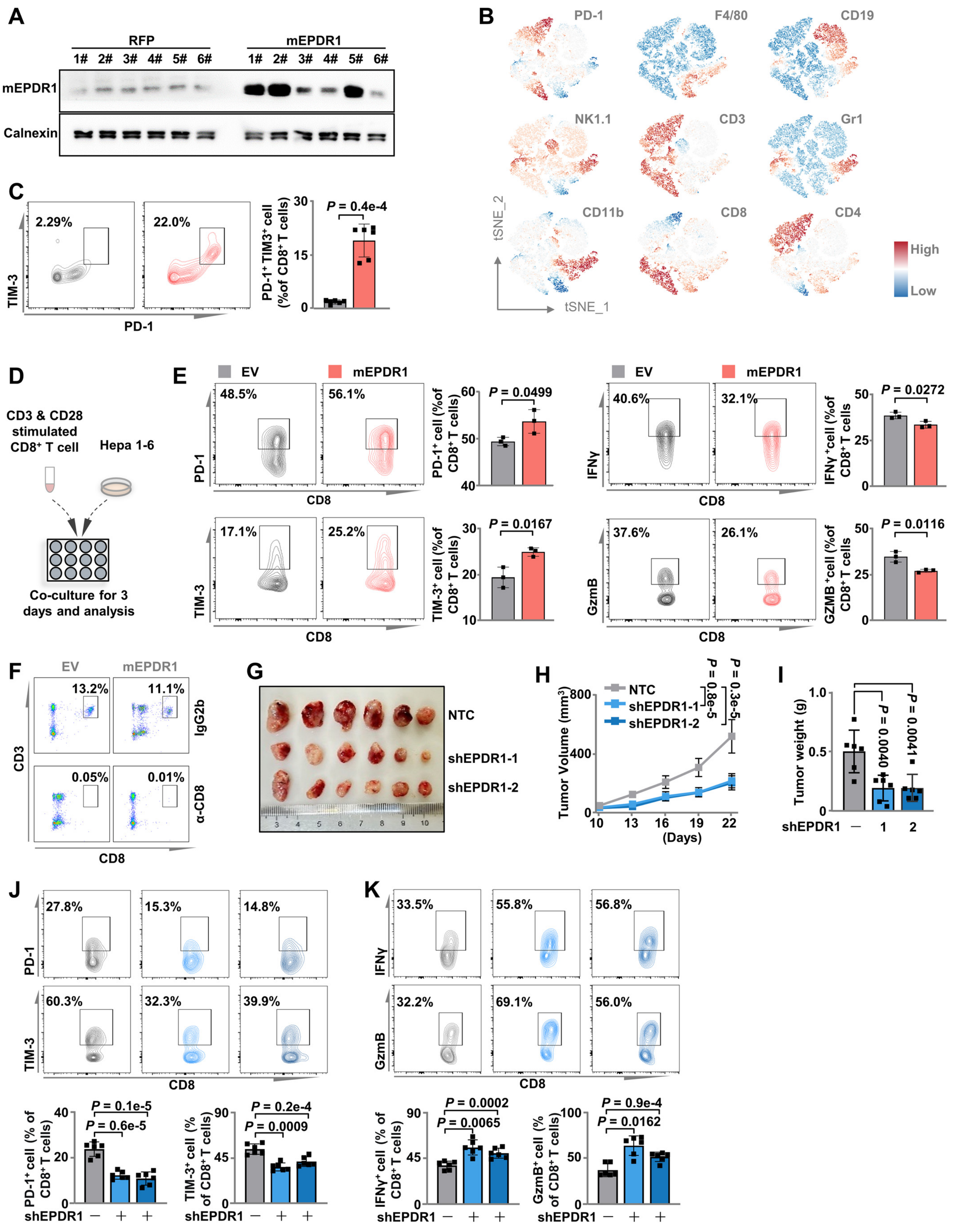
A
RFP
mEPDR1
1# 2# 3# 4# 5# 6#
1# 2# 3# 4# 5# 6#
mEPDR1
Calnexin
B
PD-1
F4/80
CD19
NK1.1
CD3
Gr1
CD11b
CD8
CD4
tSNE_2
tSNE_1
High
Low
C
2.29%
22.0%
TIM-3
PD-1
PD-1+ TIM3+ cell (%of CD8+ T cells)
P = 0.4e-4
D
CD3 & CD28 stimulated CD8+ T cell
Hepa 1-6
Co-culture for 3 days and analysis
E
EV
mEPDR1
48.5%
56.1%
PD-1
CD8
PD-1+ cell (%of CD8+ T cells)
P = 0.0499
17.1%
25.2%
TIM-3
TIM-3+ cell (%of CD8+ T cells)
P = 0.0167
40.6%
32.1%
IFNγ
IFNγ+cell (%of CD8+ T cells)
P = 0.0272
37.6%
26.1%
GzmB
GZMB+cell (%of CD8+ T cells)
P = 0.0116
F
EV
mEPDR1
13.2%
11.1%
IgG2b
0.05%
0.01%
α-CD8
CD3
CD8
G
NTC
shEPDR1-1
shEPDR1-2
H
NTC
shEPDR1-1
shEPDR1-2
Tumor Volume (mm3)
(Days)
P = 0.8e-5
P = 0.3e-5
I
Tumor weight (g)
P = 0.0040
P = 0.0041
shEPDR1 — 1 2
J
27.8%
15.3%
14.8%
PD-1
60.3%
32.3%
39.9%
TIM-3
CD8
PD-1+ cell (% of CD8+ T cells)
P = 0.1e-5
P = 0.6e-5
TIM-3+ cell (% of CD8+ T cells)
P = 0.2e-4
P = 0.0009
shEPDR1 — + +
K
33.5%
55.8%
56.8%
IFNγ
32.2%
69.1%
56.0%
GzmB
CD8
IFNγ+cell (% of CD8+ T cells)
P = 0.0002
P = 0.0065
GzmB+ cell (% of CD8+ T cells)
P = 0.9e-4
P = 0.0162
shEPDR1 — + +

 © The Author(s)

Figure EV2. Tumor-intrinsic EPDR1 facilitates immune evasion by increasing antitumor CD8$^+$ T cells exhaustion.

(**A**) Western blotting analysis of the protein level of Myc-tag mEPDR1 in liver samples from the YAP5SA-induced mouse model. Calnexin served as a loading control. (**B**) t-SNE plot showing the expression level marker genes for distinct subpopulations of lymphocytes. (**C**) Flow cytometric analysis of the ratio of immunosuppressive molecules (PD-1$^+$ TIM-3$^+$) cells in tumor CD8$^+$ T cells from the indicated group in (**A**). Data were presented as the mean ± SD. (**D**) Schema of coculture of mouse CD8$^+$ T cells with Hepa 1-6 cells expressing Flag-EV or Flag-mEPDR1. (**E**) Flow cytometry analysis of the ratio of immunosuppressive molecules (PD-1, TIM-3) and immune effector molecules (IFNγ, GzmB) positive cells in CD8$^+$ T cells after coculture with the indicated tumor cells. Data were presented as the mean ± SD of three independent experiments ($n = 3$). (**F**) Flowrate analysis of the amount of CD8$^+$ T cells in blood from the mice with the indicated manipulation. (**G–I**) Hepa 1–6 cells stably expressing NTC or shEPDR1 were injected subcutaneously into C57BL/6J mice ($n = 6$ male mice per group). Tumor size was measured starting at 10 days after inoculation. The figure depicts xenografts (**A**), growth curves (**B**), and tumor weights (**C**) determined at the end of the experiment (day 25). Data were presented as the mean ± SEM (**H**), and mean ± SD (**I**), respectively. (**J**) Flow cytometric analysis of the ratio of immunosuppressive molecules (PD-1, TIM-3) positive cells in tumor CD8$^+$ T cells from the indicated group in (**G**). Data were presented as the mean ± SD ($n = 6$). (**K**) Flow cytometric analysis of the ratio of immune effector molecules (IFNγ, Granzyme B) positive cells in tumor CD8$^+$ T cells from the indicated groups in (**G**). Data were presented as the mean ± SD ($n = 6$). Data information: Statistical significance was determined by two-tailed unpaired Student's *t*-test (**C**, **E**), two-way ANOVA (**H**), and one-way ANOVA (**I–K**). Source data are available online for this figure.

© The Author(s)

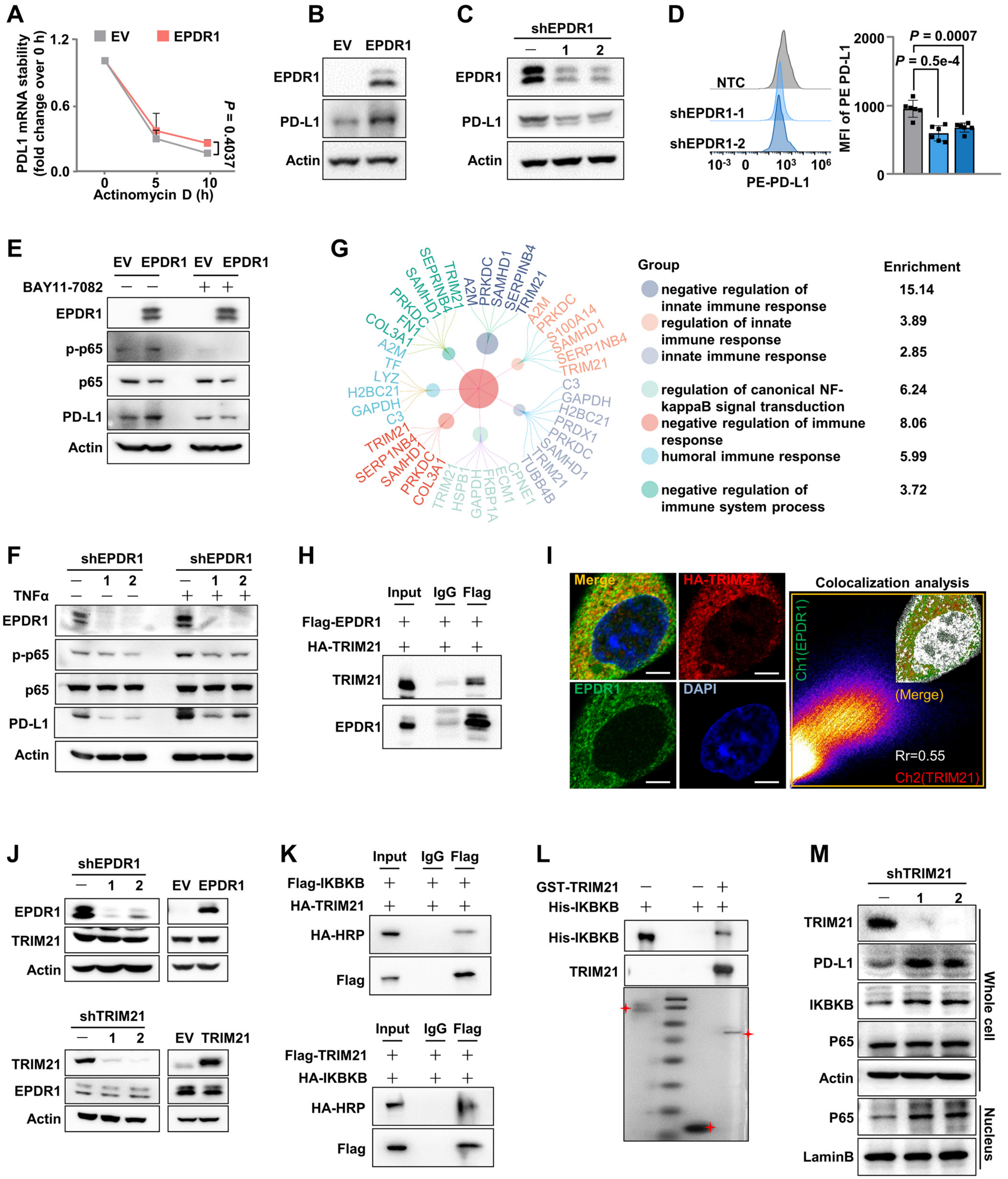
A
EV
EPDR1
PDL1 mRNA stability (fold change over 0 h)
1.2
0.6
0.0
0
5
10
Actinomycin D (h)
P = 0.4037
B
EV EPDR1
EPDR1
PD-L1
Actin
C
shEPDR1
— 1 2
EPDR1
PD-L1
Actin
D
NTC
shEPDR1-1
shEPDR1-2
10-3 0 10³ 10⁶
PE-PD-L1
MFI of PE PD-L1
2000
1000
0
P = 0.0007
P = 0.5e-4
E
EV EPDR1 EV EPDR1
BAY11-7082 — — + +
EPDR1
p-p65
p65
PD-L1
Actin
G
Group
Enrichment
negative regulation of innate immune response 15.14
regulation of innate immune response 3.89
innate immune response 2.85
regulation of canonical NF-kappaB signal transduction 6.24
negative regulation of immune response 8.06
humoral immune response 5.99
negative regulation of immune system process 3.72
F
shEPDR1 shEPDR1
— 1 2 — 1 2
TNFα — — — + + +
EPDR1
p-p65
p65
PD-L1
Actin
H
Input IgG Flag
Flag-EPDR1 + + +
HA-TRIM21 + + +
TRIM21
EPDR1
I
Merge
HA-TRIM21
EPDR1
DAPI
Colocalization analysis
Ch1(EPDR1)
(Merge)
Rr=0.55
Ch2(TRIM21)
J
shEPDR1
— 1 2
EV EPDR1
EPDR1
TRIM21
Actin
shTRIM21
— 1 2
EV TRIM21
TRIM21
EPDR1
Actin
K
Input IgG Flag
Flag-IKBKB + + +
HA-TRIM21 + + +
HA-HRP
Flag
Input IgG Flag
Flag-TRIM21 + + +
HA-IKBKB + + +
HA-HRP
Flag
L
GST-TRIM21 — — +
His-IKBKB + + +
His-IKBKB
TRIM21
M
shTRIM21
— 1 2
TRIM21
PD-L1
IKBKB
P65
Actin
P65
LaminB
Whole cell
Nucleus

 © The Author(s)

Figure EV3. EPDR1 enhances the NF-κB pathway and elevates PD-L1 expression in cancer cells by interacting with TRIM21.

(**A**) PD-L1 mRNA stability was determined in HepG2 cells expressing Flag-EV and Flag-EPDR1, cells treated with the transcription inhibitor actinomycin D (5 μM) or vehicle control for the indicated times ($n = 3$ biological replicates, data were presented as the mean ± SD, statistical significance was determined by two-way ANOVA). (**B**) Western blotting analysis of the protein levels of EPDR1 and PD-L1 in HepG2 cells expressing Flag-EV and Flag-EPDR1; β-actin was used as a loading control. (**C**) Western blotting analysis of the protein levels of EPDR1 and PD-L1 in HepG2 cells with EPDR1 knockdown; β-actin was used as a loading control. (**D**) Flow cytometry analysis of membrane binding PD-L1 on tumor cells separate from xenograft with mEPDR1 knockdown ($n = 6$ biological replicates, data are presented as the mean ± SD and statistical significance was determined by one-way ANOVA). (**E**) Western blotting analysis of the protein levels of PD-L1 and p65 in PLC cells expressing shNTC and shEPDR1. Cells were treated with the NF-κB agonist TNFα (100 nM) or vehicle control 6 h before sample collection, and β-actin was used as a loading control. (**F**) Western blotting analysis of the protein levels of PD-L1 and p65 in PLC cells expressing Flag-EV and Flag-EPDR1. Cells were treated with the NF-κB inhibitor BAY11-7082 (5 μM) or vehicle control 6 h before sample collection, and β-actin was used as a loading control. (**G**) Hierarchical network diagram showing the immune relate process that EPDR1 potential interactors involved in. (**H**) Co-IP assay showing the protein interaction between EPDR1 and TRIM21. HEK293T cells were transfected with Flag-EPDR1 and HA-TRIM21 plasmids. Cell lysates were immunoprecipitated with an anti-Flag antibody, followed by Western blotting analysis with antibodies against HA. (**I**) Representative images of immunofluorescence staining for endogenous EPDR1 and HA-TRIM21 in HepG2 cells. The nucleus was stained with DAPI; Scale bar, 20 μm. *R* were calculated by Person's correlation analysis. (**J**) Western blotting analysis of the protein expression of EPDR1 or TRIM21 in HepG2 cells with the indicated genotypes. β-Actin was used as a loading control. (**K**) Co-IP assay showing the protein interaction between IKBKB and TRIM21. HEK293T cells were transfected with Flag-IKBKB plus HA-TRIM21 or Flag-TRIM21 plus HA-IKBKB plasmids. Cell lysates were immunoprecipitated with an anti-Flag antibody, followed by Western blotting analysis with antibodies against HA. (**L**) Pull-down assay showing the protein interaction between GST-TRIM21 and IKBKB-His. GST-tagged TRIM21 and 6× His-tagged IKBKB proteins were purified from *E. coli* and incubated in vitro, followed by Western blotting analysis with antibodies against TRIM21 or IKBKB. The red asterisks indicate the target bands. (**M**) Western blotting analysis of the protein levels of PD-L1, IKBKB, and p65 in whole cell lysate and nuclear fractions in HepG2 cells expressing shNTC and shTRIM21 with GAPDH and Lamin B, as the loading controls, respectively. Source data are available online for this figure.

© The Author(s)

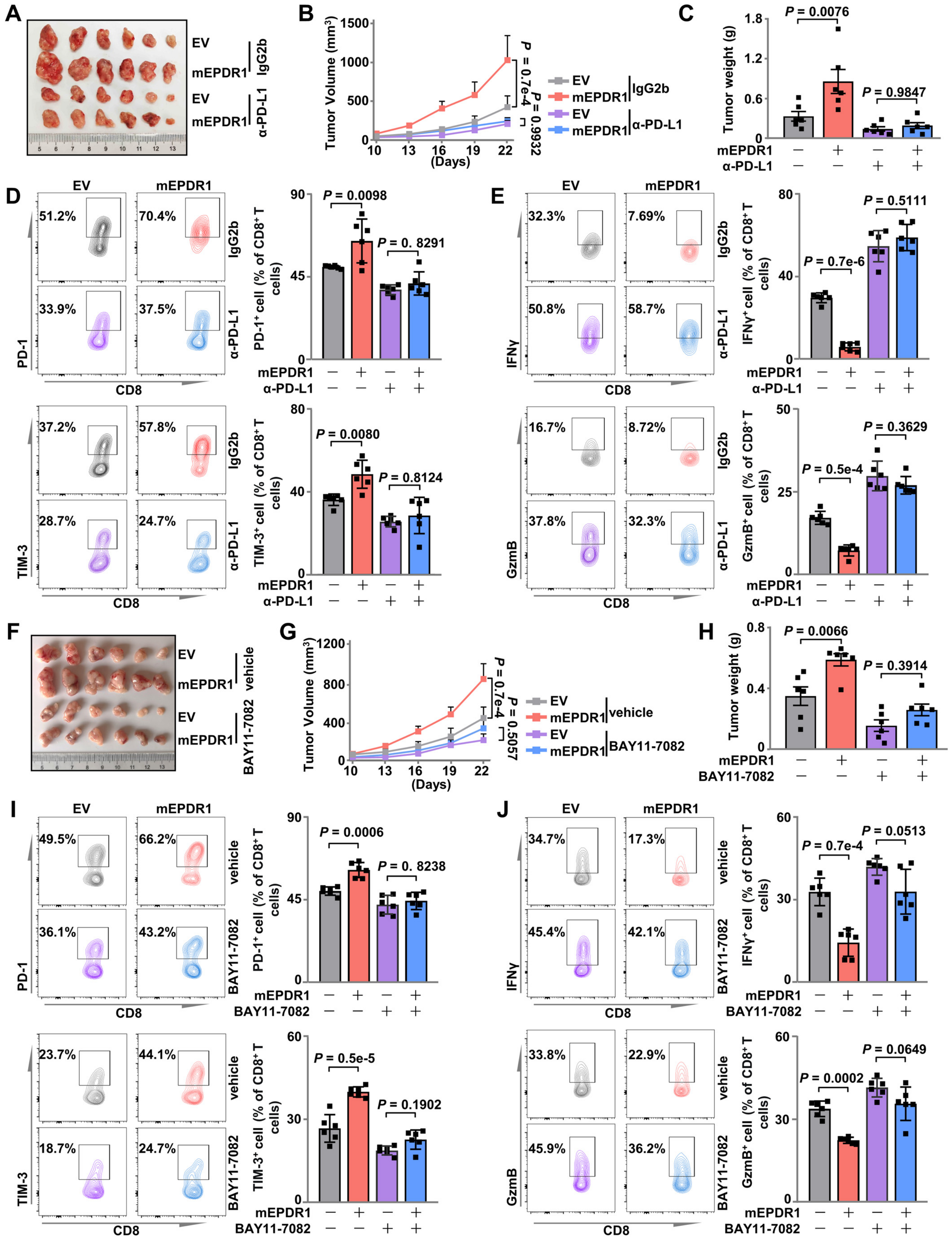

 © The Author(s)

Figure EV4. The EPDR1-TRIM21-PD-L1 axis promotes antitumor immune evasion in HCC.

(**A–C**) Hepa 1-6 cells stably expressing Flag-EV and Flag-mEPDR1 were injected subcutaneously into C57BL/6J mice ($n = 6$ male mice per group), and α-PD-L1 (6 mg/kg) neutralizing antibody was injected intraperitoneally four times (twice a week starting at 10 days after inoculation) to block PD-L1 and IgG2b was used as control. Tumor size was measured starting at 10 days after inoculation. Photographs show xenografts (**A**), growth curves (**B**), and tumor weight (**C**) determined at the end of the experiment (day 25). Data were presented as the mean ± SEM (**B**) and mean ± SD (**C**), respectively. (**D**) Flow cytometry analysis of ratio of immunosuppressive molecules (PD-1, TIM-3) positive cells in tumor $CD8^+$ T cells from the indicated group in (**A**). Data were presented as the mean ± SD. (**E**) Flow cytometry analysis of the ratio of immune effector molecules (IFNγ, Granzyme B) positive cells in tumor $CD8^+$ T cells from the indicated groups in (**A**). Data were presented as the mean ± SD. (**F–H**) Hepa 1–6 cells stably expressing Flag-EV and Flag-mEPDR1 were injected subcutaneously into C57BL/6J mice ($n = 6$ male mice per group). BAY11-7082 was used to inhibit the NF-Kb pathway, and the vehicle was used as a control. Tumor size was measured starting at 10 days after inoculation. Photographs show xenografts (**F**), growth curves (**G**), and final tumor weight (**H**) determined at the end of the experiment (day 25). Data were presented as the mean ± SEM (**G**), and mean ± SD (**H**), respectively. (**I**) Flow cytometry analysis of the ratio of immunosuppressive molecules (PD-1, TIM-3) positive cells in tumor $CD8^+$ T cells from the indicated group in (**F**). Data were presented as the mean ± SD. (**J**) Flow cytometry analysis of the ratio of immune effector molecules (IFNγ, GzmB) positive cells in tumor $CD8^+$ T cells from the indicated group in (**F**). Data were presented as the mean ± SD. Data information: Statistical significance was determined by two-way ANOVA (**B**, **G**) and one-way ANOVA (**C–E**, **H–J**). Source data are available online for this figure.

© The Author(s)

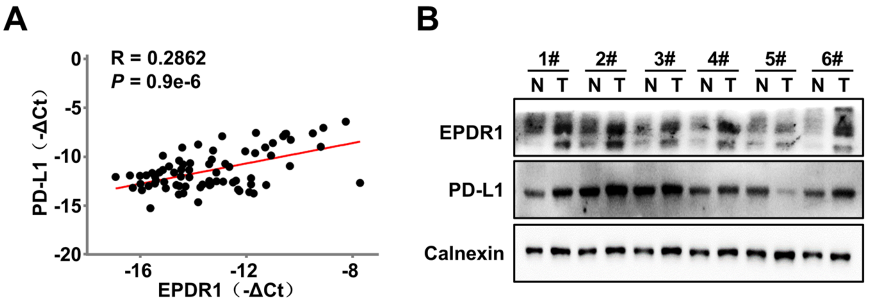

Figure EV5. The level of EPDR1 expression is positively correlated with PD-L1 transcription in HCC tissues.

(**A**) qRT-PCR analysis of the correlation of EPDR1 with PD-L1 in 20 pairs of clinically matched adjacent noncancerous liver tissues (Normal) and human liver cancer tissues (Tumor), *P* values and R were calculated by two-tailed Person's correlation analysis. (**B**) Western blotting analysis of the correlation of EPDR1 with PD-L1 in six pairs of matched adjacent noncancerous liver tissues (Normal) and mouse liver cancer tissues (Tumor). Source data are available online for this figure.
